# Current use of virtual reality in medical education in Germany, Austria, and Switzerland: Results of an online survey among medical faculties

**DOI:** 10.3205/zma001785

**Published:** 2025-11-17

**Authors:** Marie-Christin Willemer, Marcel Meyerheim, Marvin Mergen, Henriette Schulze, Tanja Joan Eiler, Lukas Mayer, Bernd F. M. Romeike, Ole Hätscher, Robert Speidel, Anna Junga

**Affiliations:** 1 TUD Dresden University of Technology, Carl Gustav Carus Faculty of Medicine, Institute of Medical Education, Medical Interprofessional Training Center (MITZ), Dresden, Germany; 2 Saarland University, Department of Paediatric Oncology and Haematology, Homburg, Germany; 3 University of Münster, Institute of Education and Student Affairs, Münster, Germany; 4 University of Siegen, Faculty of Life Sciences, Department of Psychology, Siegen, Germany; 5 Flensburg University of Applied Sciences, Center for Interaction, Visualization and Usability (CIVU), Flensburg, Germany; 6 University Medical Center Rostock, Dean's Office for Student’s Affairs, Division of Medical Education, Rostock, Germany; 7 University of Münster, Department of Psychological Assessment and Personality Psychology, Münster, Germany; 8 University of Ulm, Faculty of Medicine, Office of the Dean of Studies, Competence Center eEducation in Medicine, Ulm, Germany

**Keywords:** medical education, virtual reality, educational technology, immersive learning environments

## Abstract

**Introduction:**

Virtual Reality (VR) is playing an increasingly important role in medical education and training by enabling realistic and immersive learning environments. However, a comprehensive overview of how VR is implemented at medical faculties in German-speaking countries (“DACH region”) is still lacking. This article aims to close this gap by providing an overview of the current use of VR at medical faculties in the region, with a particular focus on immersive VR applications and the use of Head-Mounted Displays (HMDs).

**Methods:**

To investigate the use of VR, an online survey was sent to 53 medical faculties in the DACH region. The questionnaire, which was created by a consensus of experts, collected data on VR use, technical implementation and financing in addition to demographic information. The data were analysed descriptively using SPSS.

**Results:**

A total of 36 faculties (68% of DACH institutions) participated in the survey. 56% already use HMD-based VR in teaching, 11% are planning to do so. The most common goals of using VR include preparation for clinical practice and training in procedures or rare and high-risk situations. Funding sources include third-party and institutional funding, with commercially licensed applications being the most commonly used software. Technical implementation varies, and VR headsets are mostly used in specially adapted rooms.

**Discussion:**

The spread of VR is inhomogeneous. The main challenges relate to financing, technical infrastructure and concerns about data protection. There is currently no standardised and accessible platform for the networking and visibility of VR projects in the DACH region.

**Conclusion:**

Just over half of the faculties surveyed are already using VR, while others are planning to implement it. Networking and standardized evaluations are necessary to establish VR in medical teaching. The VR Working Group (VR-AG) of the DACH Association for Medical Education (GMA) provides a useful basis for this. In addition, further studies are needed to evaluate the long-term learning effects and practical applicability of VR applications.

## Zusammenfassung

**Einleitung:**

Virtual Reality (VR) wird zunehmend in der medizinischen Aus- und Weiterbildung genutzt, um realitätsnahe immersive Lernumgebungen zu schaffen. Der Überblick über den Einsatz von VR an medizinischen Fakultäten im deutschsprachigen Raum (DACH-Region) ist bisher unzureichend.

Ziel dieses Artikels ist es, einen entsprechenden Überblick über den Einsatz von VR an medizinischen Fakultäten in dieser Region zu geben, mit Fokus auf immersive VR und Head-Mounted Displays (HMDs).

**Methoden:**

Zur Erhebung des VR-Einsatzes wurde eine Onlinebefragung an 53 medizinische Fakultäten in der DACH-Region versandt. Der Fragebogen, der durch ein Expert:innen-Konsens erstellt wurde, erfasste neben demografischen Angaben Daten zur VR-Nutzung, technischen Umsetzung und Finanzierung. Die Daten wurden deskriptiv in SPSS analysiert.

**Ergebnisse:**

Insgesamt nahmen 36 Fakultäten (68% der DACH-Standorte) an der Befragung teil. 56% nutzen bereits HMD-gestützte VR in der Lehre, 11% planen dies. Die häufigsten Ziele des VR-Einsatzes umfassen die Vorbereitung auf den klinischen Einsatz sowie das Training von Abläufen oder seltenen und risikoreichen Situationen. Finanzierungsquellen umfassen Drittmittel und Institutionengelder, wobei kommerzielle Lizenzen die am häufigsten verwendete Software darstellen. Die technische Umsetzung variiert, und der Einsatz erfolgt überwiegend in speziell dafür optimierten Räumen.

**Diskussion:**

Die Verbreitung von VR ist inhomogen. Herausforderungen betreffen vor allem die Finanzierung, technische Infrastruktur und Datenschutzbedenken. Für die Vernetzung und Sichtbarkeit von VR-Projekten in der DACH-Region gibt es bislang keine einheitliche und zugängliche Plattform.

**Schlussfolgerung:**

Etwas über die Hälfte der befragten Fakultäten setzten VR bereits ein, weitere planen einen Einsatz. Um VR in der medizinischen Lehre zu etablieren sind Vernetzung und standardisierte Evaluationen nötig. Die VR-Arbeitsgruppe der Gesellschaft Medizinische Ausbildung (GMA) bietet hierfür eine sinnvolle Grundlage. Zudem sind weitere Studien notwendig, um den langfristigen Lerneffekt und die praktische Anwendbarkeit von VR-Anwendungen zu bewerten.

## 1. Introduction

The integration of Virtual Reality (VR) into medical education and training has gained increasing importance in recent years. VR offers an innovative opportunity to impart medical knowledge, train skills and promote competencies through immersive and interactive environments [1].

VR is particularly suitable as a learning scenario in dangerous or rare situations, as these are otherwise difficult to simulate [2], [3], [4]. Even topics that are difficult to approach (e.g. intimate examinations, post-mortem examination) or rare diseases can be taught in a practical way. VR also enables asynchronous learning independent of teaching staff, which can save resources [5], [6], [7].

In addition to practising operational skills, Non-Technical Skills (NTS) such as communication, critical thinking and clinical decision-making are also trained in VR [8]. For educational research, VR offers a simple way of modelling and analysing social and cognitive behaviours [9].

Scientific approaches examining the learning impact of VR applications are receiving increasing attention. The results of studies have shown that training sessions in VR lead to an improvement in surgical skills in the operating theatre [10]. A systematic review and a meta-analysis show positive effects of VR applications on learning and student performance as well as their superiority over conventional teaching methods [11], [12].

Medical students also see great benefits in VR, particularly in surgery, emergency medicine and anatomy. They would like to see it integrated into the medical curriculum [13], [14].

Although there are international overview articles on areas of application, e.g. [15], [16], [17], and the range of German-language VR software has increased [18], [19], [20], an overview of university and commercial VR applications in German-speaking region (DACH region) has been lacking so far. The aim of this study is to assess and descriptively present the use and technical implementation of VR at medical faculties in the DACH region through an online survey. The focus is on immersive VR and head-mounted displays (HMDs). Although there are already efforts in other healthcare professions to include VR in training [21], [22], this initial survey should primarily look at the distribution at medical faculties. An expansion of this can be addressed in a follow-up study.

## 2. Method

### 2.1. Development of the questionnaire

To capture the current use of VR in medical teaching at the various medical faculties in the DACH region, an online survey was conducted using a questionnaire. The questionnaire was created by a focus group consisting of members of the interdisciplinary VR Working Group (VR-AG) of the GMA-committee for “Digitisation – Technology-supported Learning and Teaching” of the DACH Association for Medical Education (GMA), which was founded in March 2022. The development of the questionnaire went through several rounds of feedback to take all relevant aspects into account and to ensure that the questions were uniformly comprehensible. The questionnaire (see attachment 1 , only in German) covered the following areas:

- Personal and location-related information provided by respondents
- General information on the use of VR
  - Current status
  - Reasons for implementation
  - Intended goals
- Technical implementation
- Didactic scenarios
- VR research areas and networks
- Funding and promotion of VR

The use of VR focused on immersive applications in which Head-Mounted Displays (HMDs) are used. VR headsets using smartphones as displays were excluded, which was referred to in the questionnaire. Depending on the type of VR use, up to 45 questions could be answered. The questions were formulated as closed and open questions, with single-choice, multiple-choice and free-text response formats. They were then transferred to the online survey platform LimeSurvey [https://www.limesurvey.org/de] and made available as a link and via QR code.

### 2.2. Collection of data

The online survey was launched in April 2023. A cover letter with access to the questionnaire and the objective of the status quo survey was emailed to 50 medical faculties in German-speaking countries: 41 in Germany, 5 in Austria and 4 in Switzerland. In addition, a call for participation including a link to the survey was published in the GMA newsletter in April 2023, accessible to all universities. The data collection was completed in June 2023. Participation in the survey was voluntary.

### 2.3. Data processing and analysis

The data were transferred to SPSS Statistics Version 29.0.0. (International Business Machines, IBM, Armonk, New York, USA). Multiple responses from individual faculties were combined. The responses were analysed using descriptive statistics and evaluated in terms of both absolute and relative frequencies. Personal details of the respondents were only used to ask questions if necessary.

## 3. Results

### 3.1. Response rate

**Figure 1:**
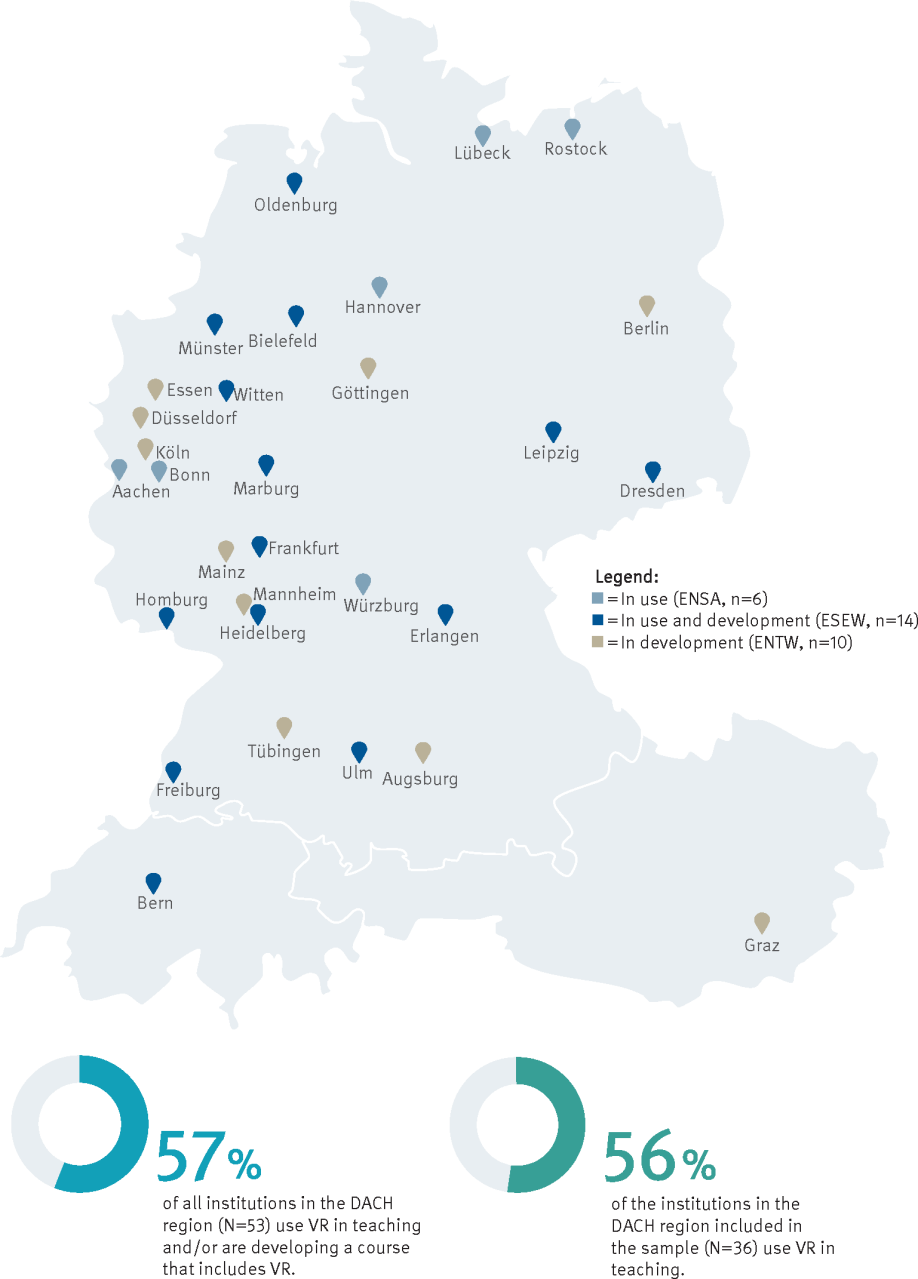
Use and Development of VR in medical education in the DACH region ENSA=In use without development, ESEW=In use and development, ENTW=In development without prior use

 A total of 53 responses were received in the study, 5 of which did not contain any location-specific or other information and were therefore removed from the data collected. Of the 48 remaining responses, some were summarized because several locations (Bern, Dresden, Freiburg, Göttingen, Hanover, Heidelberg, Lübeck, Münster, and Witten) submitted a few individual project responses, which we combined as described in the Methods section. Ultimately, feedback from 68% (36 of 53) of the DACH locations offering medical studies could be evaluated (see figure 1 (Fig. 1)). The responses cover 73% (32 out of 44) of the locations in Germany, 40% (2 out of 5) of the locations in Austria and 50% (2 out of 4) of the locations in Switzerland.

### 3.2. Status of Implementation

As shown in figure 1 (Fig. 1), 56% (20 out of 36) of the locations stated that they already use HMD-supported VR applications in medical education. These 20 locations can be divided into 2 groups: 6 locations are using VR in teaching but are not currently developing any new courses with VR components (in use without development: **E**i**NSAt**z ohne Entwicklung – [ENSA]) and 14 locations are both using and developing VR-based courses (in use and development: Standort mit **E**in**S**atz & **E**nt**W**icklung von VR – [ESEW]). In addition, 28% (10 out of 36) of the locations are not yet using VR in teaching but are developing corresponding courses (in development without prior use: **ENTW**icklung ohne bisherigen Einsatz – [ENTW]). The remaining 16% (6 out of 36) are neither using VR nor developing it, but 4 of these plans to use HMD-supported VR in teaching in the future (**no VR**: NOVR). A lack of funding or an unfavourable cost-benefit ratio were mentioned three times as reasons for not planning to use VR.

### 3.3. Objectives and reasons for the use of VR in teaching

The objectives shown in table 1 (Tab. 1) and the reasons for using VR shown in table 2 (Tab. 2) include feedback on existing use (ENSA, ESEW) as well as development (ENTW) and planned use (NOVR).

In addition, objectives and reasons such as alternatives to internships during the pandemic, research on the topic of VR, earlier practical exposure, exposure to IT and technical developments, resource optimisation, preparation of students for clinical encounters, internalisation of procedures, interprofessional communication, rapid action in emergency situations and access to restricted areas were also mentioned. A digitalisation project in Baden-Württemberg was mentioned in the ENTW.

### 3.4. Funding sources

Table 3 (Tab. 3) provides an overview of funding for VR projects in medical teaching. The mentioned funding sources include institutional funds, contracts with commercial providers, and private funds that are to be replaced by third-party funds. Other sources of third-party funding include the Bundesministerium für Bildung und Forschung (BMBF; Federal Ministry of Education and Research), state ministries, and European funds. Out of 13 locations (ENSA and ESEW) reporting third-party funding for ongoing costs of VR in teaching, 62% stated that the use of VR would continue after funding. For ENTW projects, a special budget and cooperations were named as sources of funding.

### 3.5. Technical implementation

As shown in table 4 (Tab. 4), 80% of ENSA and ESEW (16 out of 20) use externally developed software, with the majority (88%) having acquired usage rights in the form of commercial licenses. One location uses Creative Commons (CC) licenses. At another location, the VR application is provided by a project coordination partner, while two other locations that use commercial software also offer self-developed or co-developed scenarios.

At the ENTW and ESEW, 58% (14 out of 24) plan to use external software in their courses with VR. Among these 14 locations, 57% will use only commercial licenses, 14% will use only CC licenses and 22% will use a mixture of both license types. One of these locations (7%), which works with cooperation partners, has not yet concluded any detailed agreements on usage rights. In total, 25% of the 24 locations had not yet taken a clear position on the use of external software at the time of the survey.

All locations involved in 3D modelling of VR software were willing to make their applications available; two thirds under a CC license, with the remaining still clarifying licensing details.

**Figure 2:**
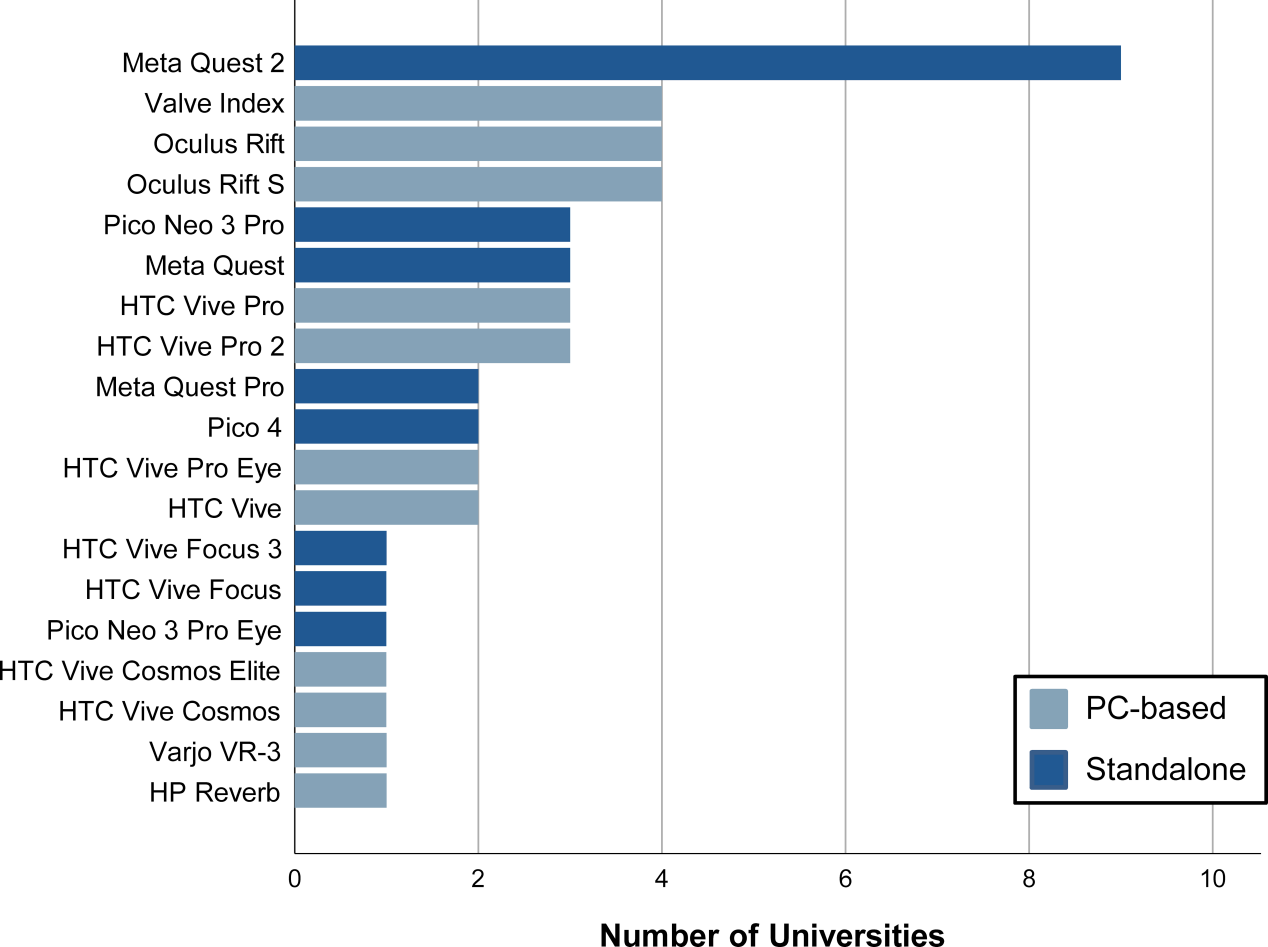
Overview of the HMDs used at locations with existing VR use (ENSA, ESEW)

**Figure 3:**
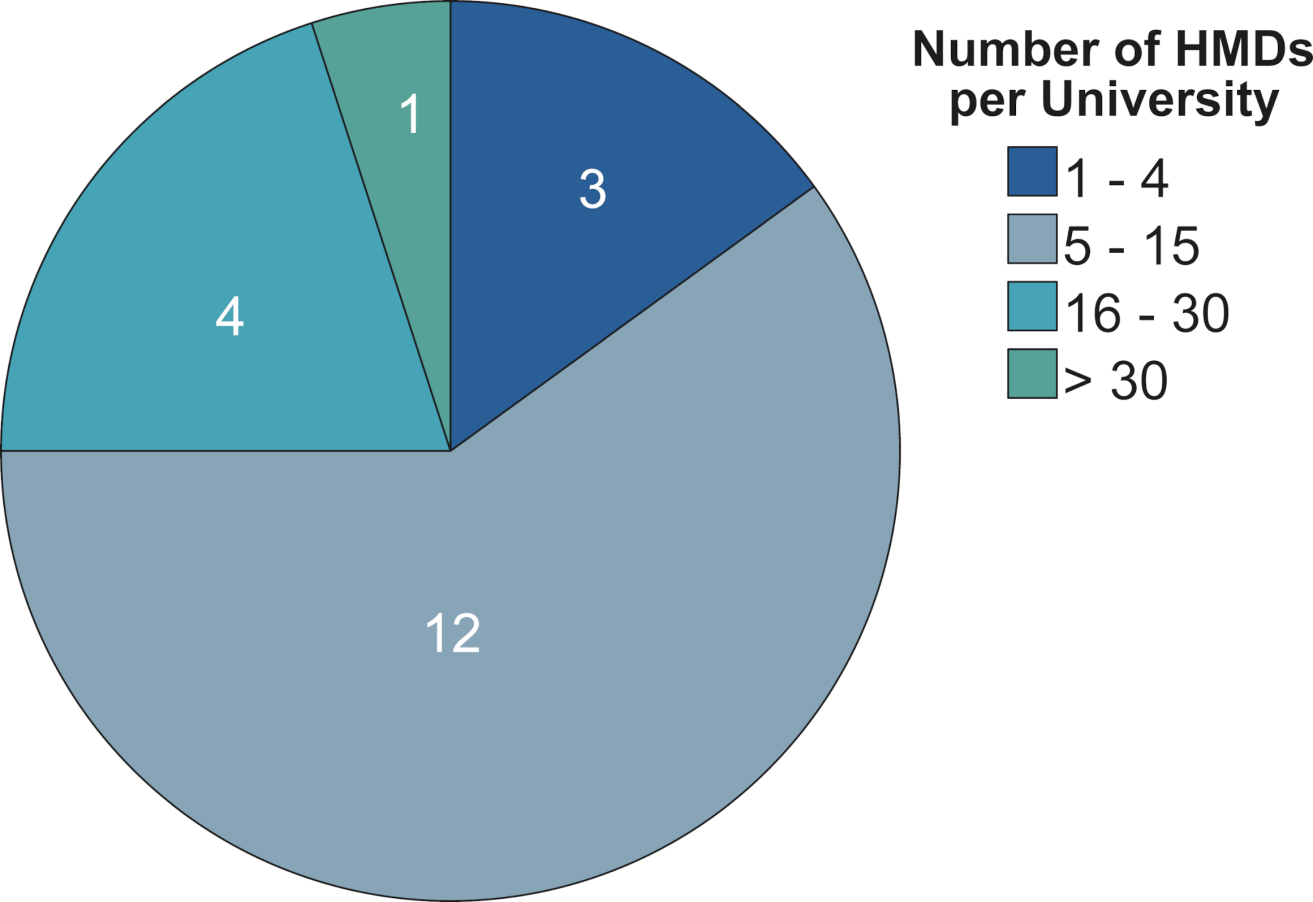
Number of HMDs used at universities with existing VR use (ENSA, ESEW)

Figure 2 (Fig. 2) shows that the Meta Quest 2 is the most commonly used HMD for existing VR use at ENSA and ESEW. 60% of these locations have at least one standalone HMD that can be used without an external computer or external tracking systems. Figure 3 (Fig. 3) shows that only one location has more than 30 HMDs. Only one location uses data gloves in addition to the controllers. No other special input and output devices are used.

With regards to the learning environment, table 5 (Tab. 5) shows that rooms specially optimised for VR are used in 65% of ENSA and ESEW and are planned for at least 50% of ENTW. Technical support for the VR HMDs at ENSA and ESEW is provided by various roles, as shown in table 6 (Tab. 6). These include digitalisation teams from the medical faculties, as well as e-learning specialists, academic staff, and industry employees. Media technology and IT departments are jointly responsible for support at ENTW. Some locations are in the testing and evaluation phase, often in cooperation with industry partners and developers.

### 3.6. Curricular integration

The use of VR in various departments is summarised in table 7 (Tab. 7). Overall, VR is mainly used in clinical subjects (partly due to the majority of clinical departments).

Table 8 (Tab. 8) shows that VR is increasingly used in ENSA and ESEW over the course of medical school, except in the last two semesters. However, this trend is less pronounced for ENTWs.

As table 9 (Tab. 9) shows, VR is mainly used in seminars, small group sessions or practical courses, while it is less frequently used in lectures or tutorials. The courses are predominantly curricular or optional, with half of them being electives.

The number of participants in the VR courses varies greatly depending on the location, as can be seen in table 10 (Tab. 10). Around half of them have up to 150 participants, while a quarter are designed for more than 250 participants per semester.

Table 11 (Tab. 11) shows that VR is used differently at different locations. One location offers VR loan devices for students to use for self-directed learning. At three ENTW locations, self-learning with VR is planned. Another ENTW location is planning a rollout following a pilot phase.

### 3.7. VR research areas and networks

As shown in table 12 (Tab. 12), more than half of the existing VR applications at ENSA and ESEW are at least partly part of funded research or cooperation projects. Three quarters associate the use of VR with a scientific objective. In contrast, more than half of the ENTWs are non-cooperative projects. In accompanying studies, the research focus is often on comparing didactic methods and in one location the acceptance of VR was mentioned as an additional focus.

Cross-location dialogue is highlighted in table 13 (Tab. 13). While one location stated that it was not aware of any exchange forums, others mentioned internal and cross-university interdisciplinary working groups on VR and Extended Reality (XR). Other opportunities for exchange include the digitalisation project in Baden-Württemberg and personal communication.

There are currently a number of ongoing VR projects, including “medical tr.AI.ning”, “Comed/VISL”, “Moleküle in der Symphonie der Sinne” (“Molecules in the symphony of the senses”), “KodiLL”, “participate@UOL”, “vr-osce”, “VR-Hirntoddiagnostik” (“VR brain death examination”), “Ai.vatar”, “DiViFaG”, “Virtual Reality in der biomedizinischen Ausbildung” (“VR in biomedical training”), “XR in BW” and “VR-Leichenschau” (“VR corpse examination”). 

### 3.8 Beginning of the use of VR in teaching 

**Figure 4:**
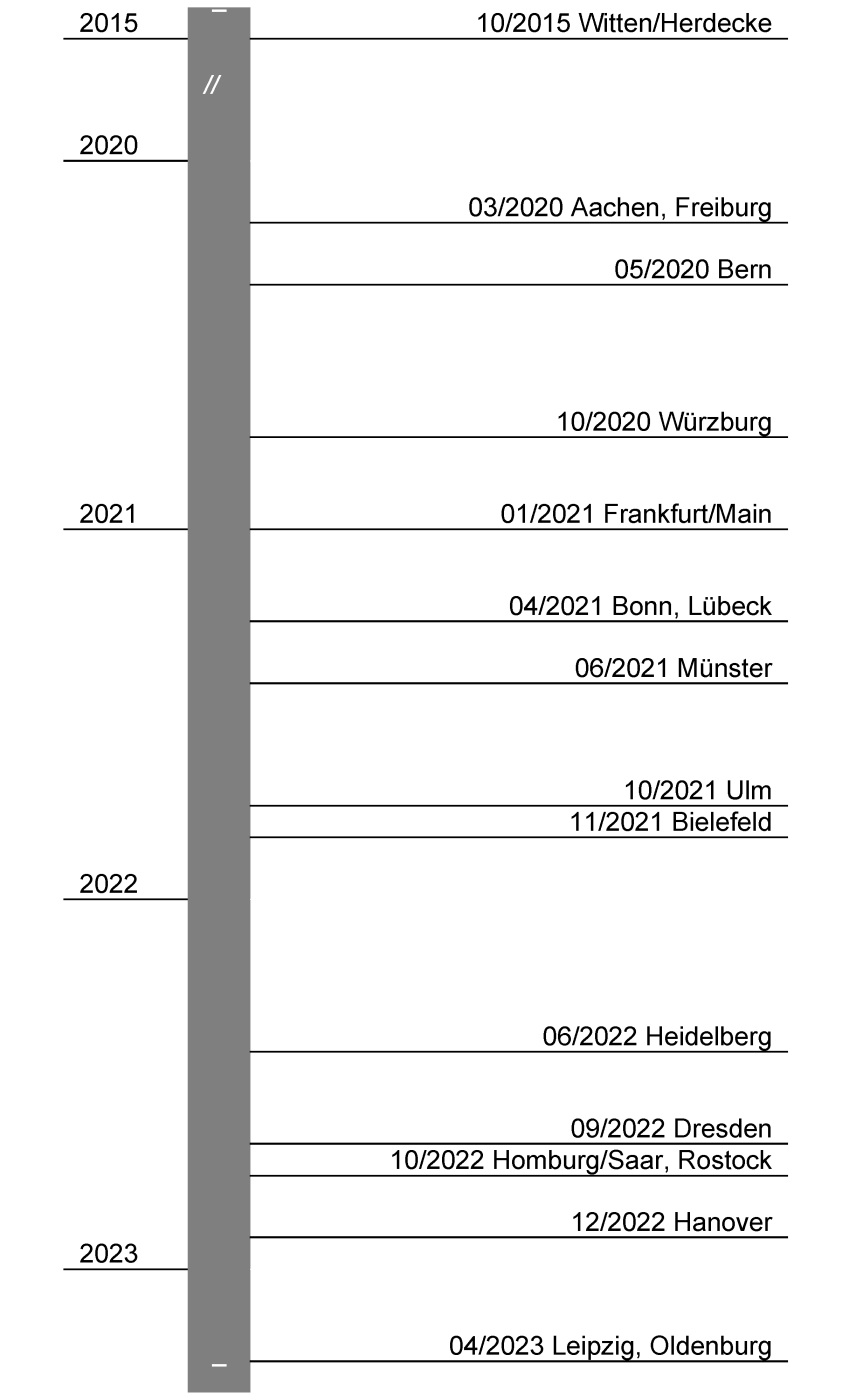
Timeline for the introduction of VR at universities in the DACH region (ENSA, ESEW)

Figure 4 (Fig. 4) provides an overview of the timeframe during which HMD-based VR applications were implemented at the respective locations.

## 4. Discussion

### 4.1. Challenges in the use of VR

The use of VR in medical education across the DACH region is inhomogeneous. While some locations use self-developed software and others use existing VR applications, around half of the universities do not use VR at all.

Challenges are primarily financial, technical and didactic in nature, such as the high costs of VR hardware, the required IT infrastructure, and the lack of technical expertise.

Despite the initially high implementation costs of VR, its long-term use can lead to cost-effectiveness [20] through savings in high personnel and training material costs (especially the costs of simulation personnel) [19], [20].

The lack of integration of haptic feedback, data protection concerns and scepticism towards new technologies also hinder wider usage [23], [24]. Data protection in particular is a major problem, as data collection and transmission by the software/hardware often cannot be deactivated in commercial applications [25].

### 4.2. Approaches to solutions

 The implementation of VR in medical training can be promoted through targeted measures such as networking via working groups, joint development projects, and standardised evaluation methods. An interactive VR-AG platform for documenting projects and further progress should create transparency and strengthen cooperation across the DACH region. Open-source solutions facilitate knowledge transfer and the adaptation of developments, which reduces the burden on universities. The exchange of teaching formats and methods should be adapted to the requirements of the institutions in a resource-saving and flexible manner, despite the challenges posed by different hardware. Successful examples include the transfer of VR post-mortem examinations and VR brain death examinations between different locations [23], [26].

### 4.3. Limitations of the survey 

Not all locations and VR applications in the DACH region could be captured in the survey, which is due to the voluntary nature of the survey or potential hurdles, such as a lack of information sharing. In addition, the respondents may not have had a complete overview of the faculty’s entire VR curriculum. The questionnaire, based on an expert consensus, did not include precise information on the number of applications, which made the analysis more difficult. Multiple responses per location were summarised and some questions remained unanswered for planned VR applications.

Although this could lead to biases, the aim of collecting information was still accomplished. The results represent a snapshot and do not take any interim developments into account. An ongoing survey is necessary to track current trends. The VR-AG of the GMA and an interactive map could provide solutions here. As the survey only covers the DACH region, the results may not be comparable internationally.

No similar data surveys exist and although international VR review articles are available, they do not provide a detailed overview of current applications in the DACH region (scoping reviews: [15], [16], [17], [27], [28], [29], [30]). Therefore, situating the findings within an international context is not appropriate.

An important step in planning and before application is to carefully review the use and appropriateness for each individual case. Various publications offer approaches and guiding questions to support the procedure [15], [17], [26]. A summary of such publications with recommendations on the use of VR in medical teaching may be a future task of the VR-AG.

### 4.4. State of research

In the relatively new field of medical training based on VR applications, there has been little research to date. A meta-analysis by Chen et al. [31] has already shown that VR clearly promotes the acquisition of knowledge, but no significant differences in skills, satisfaction, confidence or performance time were found. Further studies are needed to better understand the learning effect of VR and long-term skills development in medical education. These should not only record direct and objective learning outcomes but also shed light on the sustainable increase in knowledge and practical applicability in everyday clinical practice. More in-depth evaluations can be carried out on the basis of increased integration into the compulsory curriculum in order to minimise bias and create a representative database. Studies that examine the learning effect taking into account different learning objectives (cognitive, psychomotor, affective) could also provide valuable insights into the didactic application possibilities of VR.

## 5. Conclusion/Outlook

The use of VR in medical teaching is steadily increasing but is heterogeneous in the DACH region. External studies confirm the great potential of VR to improve learning processes and enable practical training under realistic conditions. To fully realise this potential, development work should not be carried out in isolation at individual locations but should be coordinated across locations. The current work pursues this goal: it is intended to promote the necessary exchange between the various institutions and serve as a basis for initiating joint research projects. This networking enables VR to be integrated into medical training in a targeted and effective way, while also facilitating the development of innovative teaching approaches that extend beyond individual locations.

## Abbreviations

- AG: Working Group (Arbeitsgruppe)
- BMBF: Bundesministerium für Bildung und Forschung (Federal Ministry of Education and Research)
- CC: Creative Commons
- DACH: German-speaking region; Germany: Deutschland (D), Austria: Österreich (A), Switzerland: Schweiz (CH)
- ENSA: Locations with existing VR use without development (Standorte mit bestehendem VR-**E**i**NSA**tz ohne Entwicklung)
- ENTW: Locations in development without prior use of VR (Standorte in **ENTW**icklung ohne bisherigen Einsatz von VR)
- ESEW: Locations with both use and development (Standorte mit **E**in**S**atz & **E**nt**W**icklung von VR)
- GMA: DACH Association for Medical Education (Gesellschaft für Medizinische Ausbildung)
- HMD: Head-Mounted Display
- MBO-Ä: Model Professional Code of Conduct for Doctors Practising in Germany (Muster-Berufsordnung für die in Deutschland tätigen Ärztinnen und Ärzte)
- NKLM: National Competence-Based Learning Objectives Catalogue for Medicine (Nationaler Kompetenzbasierter Lernzielkatalog Medizin)
- NOVR: Locations without (no) VR
- NTS: Non-Technical Skills
- PJ: Practical Year (Praktisches Jahr)
- VR: Virtual Reality
- XR: Extended Reality

## Notes

### Author contributions

- Project administration: MW, RS, AJ
- Conceptualisation: MW, MMey, HS, TE, RS
- Methods: MW, MMey, MMer, HS, TE, LM, BR, OH, RS, AJ
- Formal Analysis: MMey, RS, OH, LM
- Graphics and figures: OH, LM, MMey, BR
- Writing – Manuscript: MW, MMey, MMer, HS, TE, LM, BR, OH, RS, AJ
- Proof and Correction: MW, MMey, MMer, HS, TE, LM, BR, OH, RS, AJ

All authors read and approved the final manuscript and translation.

### Authors’ ORCIDs

- Marie-Christin Willemer: [0009-0000-7950-5922]
- Marcel Meyerheim: [0000-0002-9294-9445]
- Marvin Mergen: [0000-0002-3891-580X]
- Henriette Schulze: [0009-0001-4364-7141]
- Tanja Joan Eiler: [0000-0002-6917-7942]
- Lukas Mayer: [0009-0008-0261-7932]
- Bernd F. M. Romeike: [0000-0002-9693-3870]
- Ole Hätscher: [0009-0009-1410-4023]
- Robert Speidel: [0000-0001-5488-7100]
- Anna Junga: [0000-0002-4165-9114]

### Ethics

The final questionnaire was submitted to the responsible ethics committee for the medical faculty of the University of Ulm in accordance with the requirements of the ethics application together with the data protection review of the declaration of consent and data protection information. According to § 15 MBO-Ä, the survey is not subject to consultation in the sense of a teaching evaluation.

### Data availability

The anonymised data sets generated and analysed for this study can be provided by the corresponding author upon reasonable request.

## Acknowledgements

We thank all faculties for participation.

## Competing interests

The authors declare that they have no competing interests. 

## Supplementary Material

Questionnaire (only in German)

## Figures and Tables

**Table 1:**
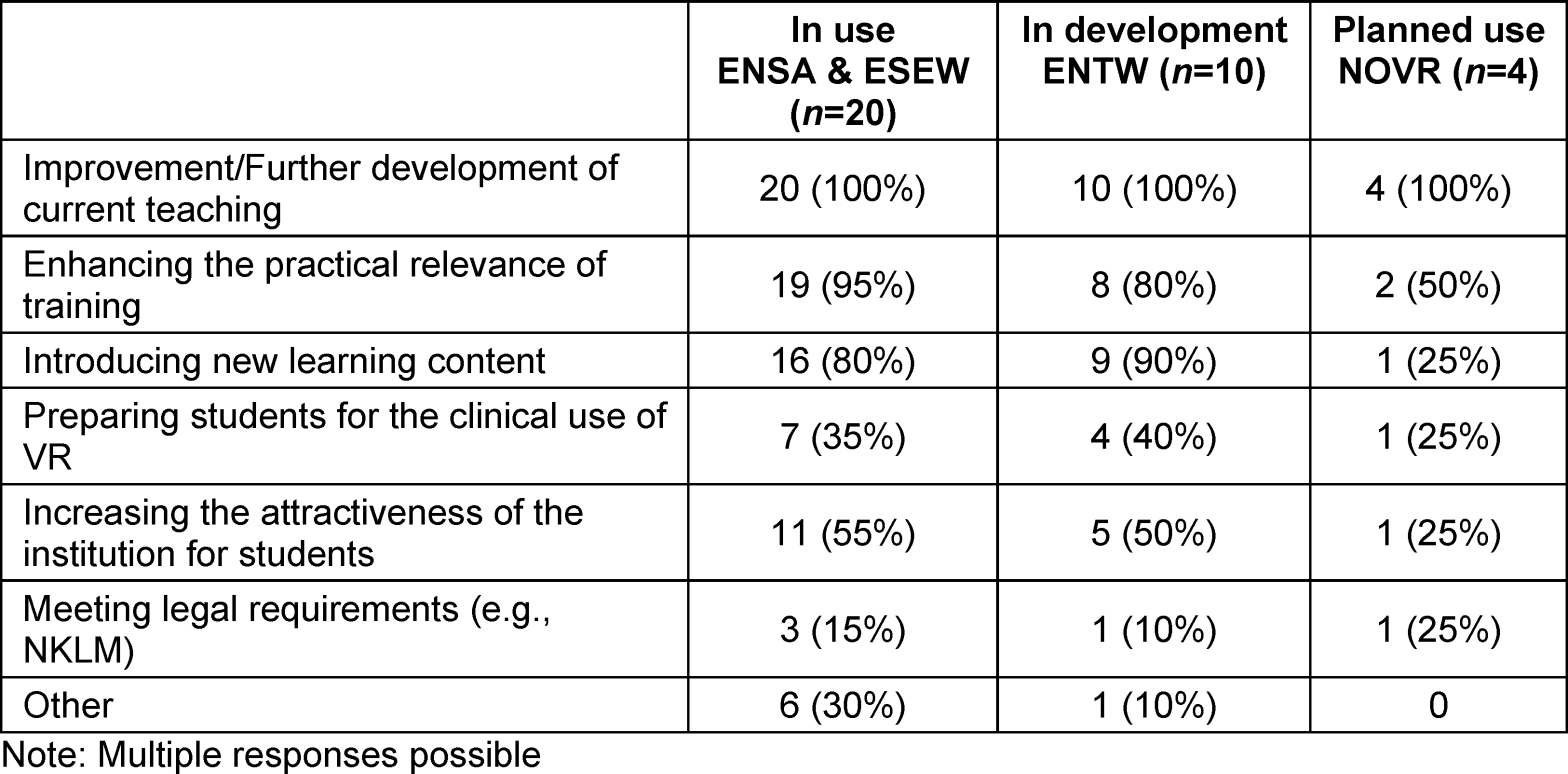
Objectives for using VR in teaching

**Table 2:**
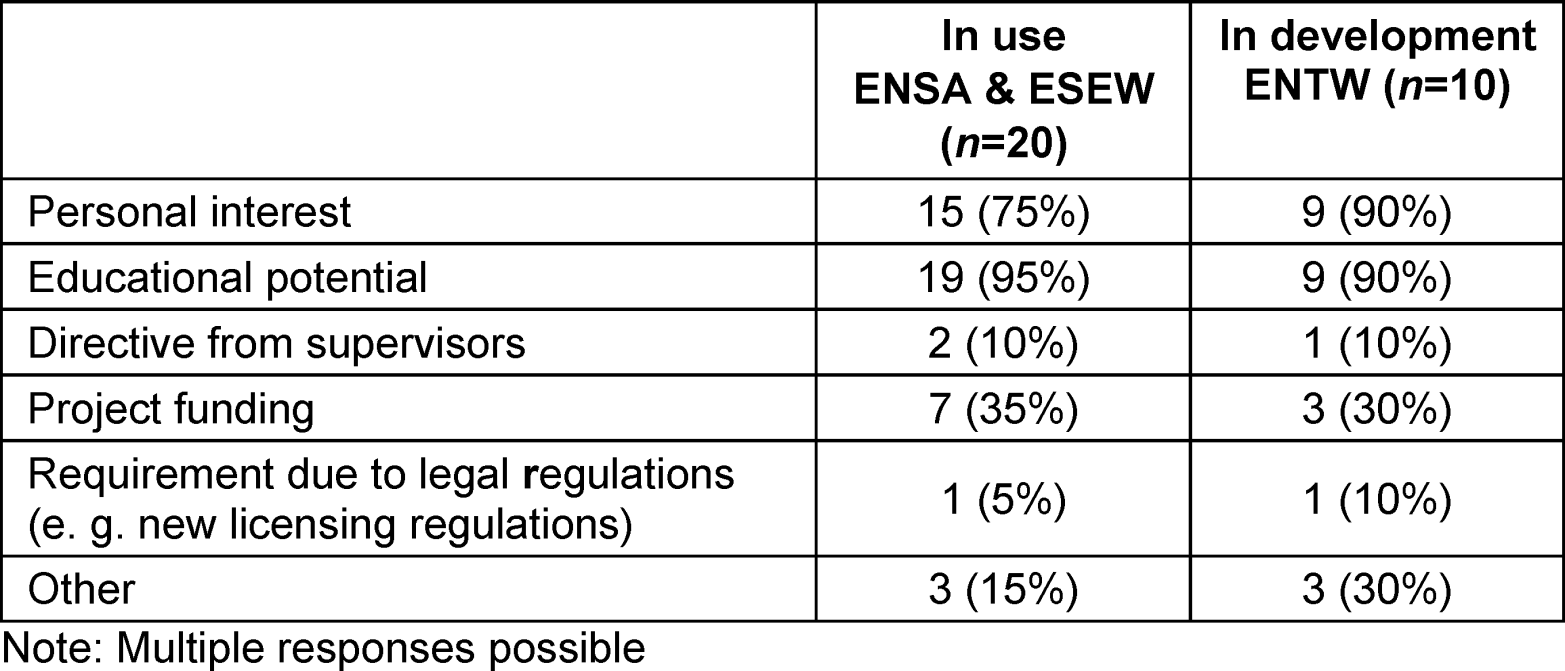
Reasons for using VR in teaching

**Table 3:**
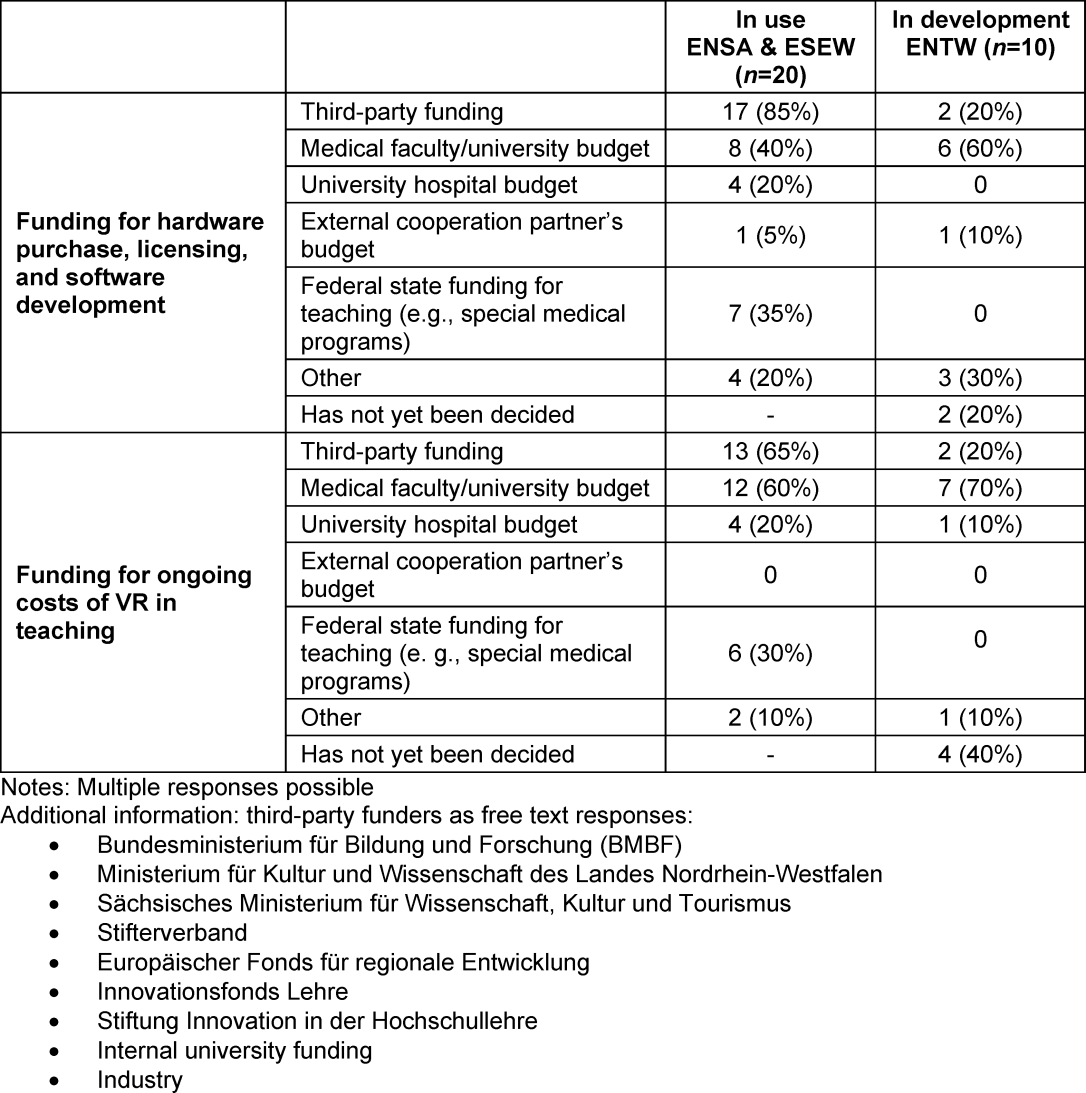
Funding sources for costs related to the use/development of VR in teaching

**Table 4:**
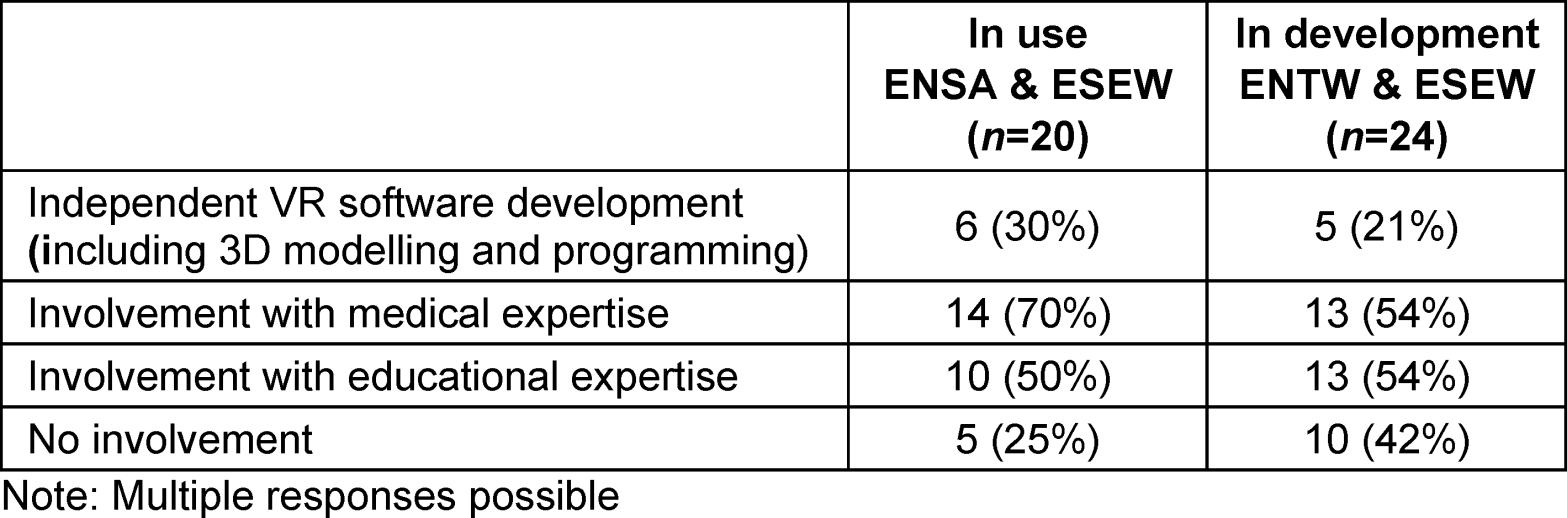
Involvement of locations in the development of VR software/applications already in use or in development

**Table 5:**
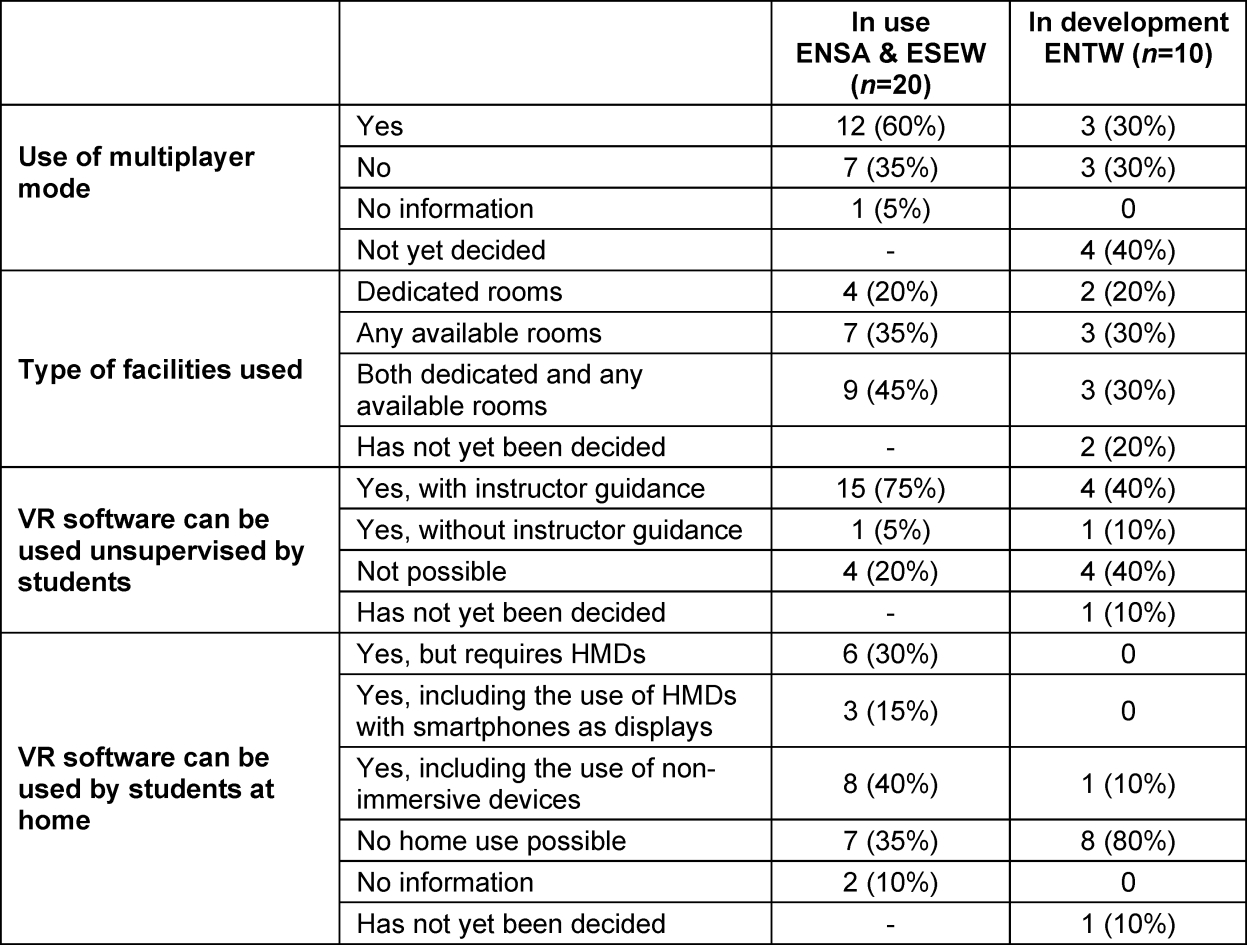
Facilities and usage of VR software

**Table 6:**
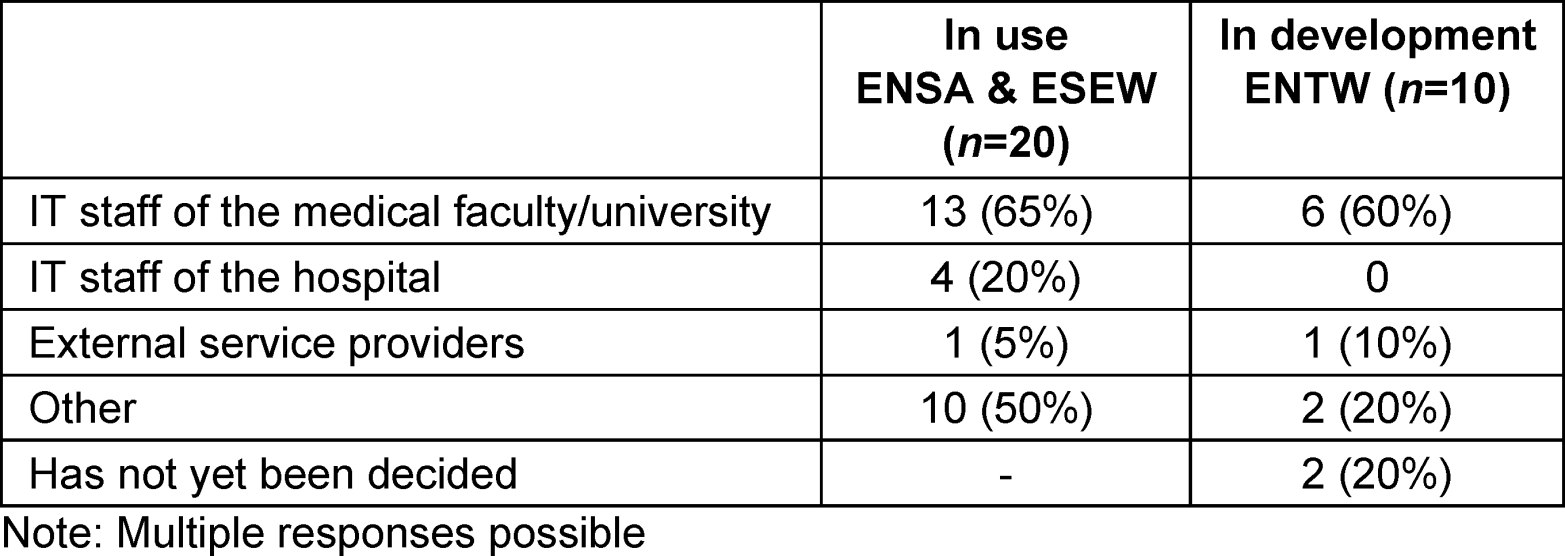
Responsible parties for technical support, maintenance, and network integration of hardware

**Table 7:**
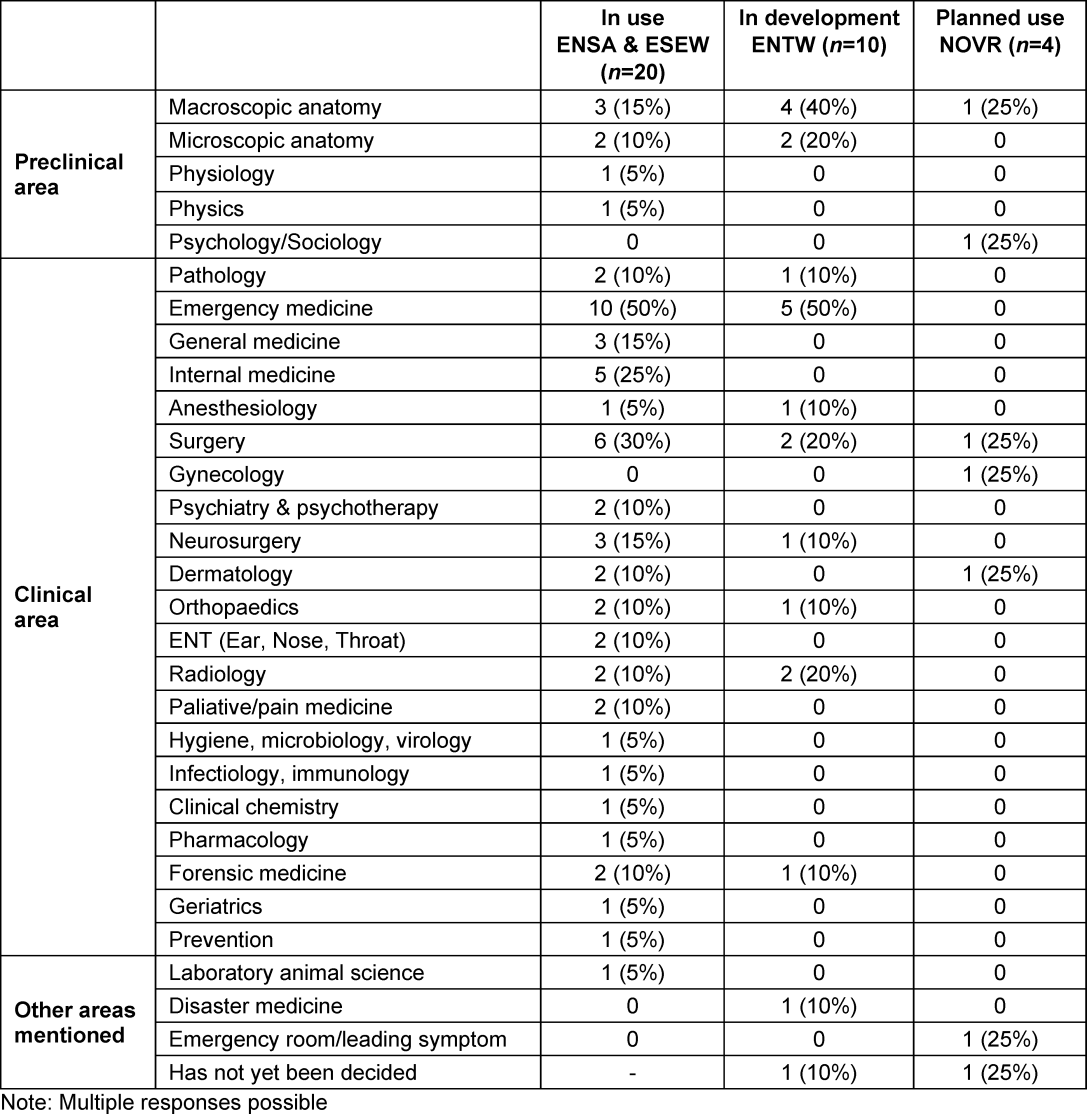
Specified medical specialties in which courses with VR components are already in use, in development, or planned

**Table 8:**
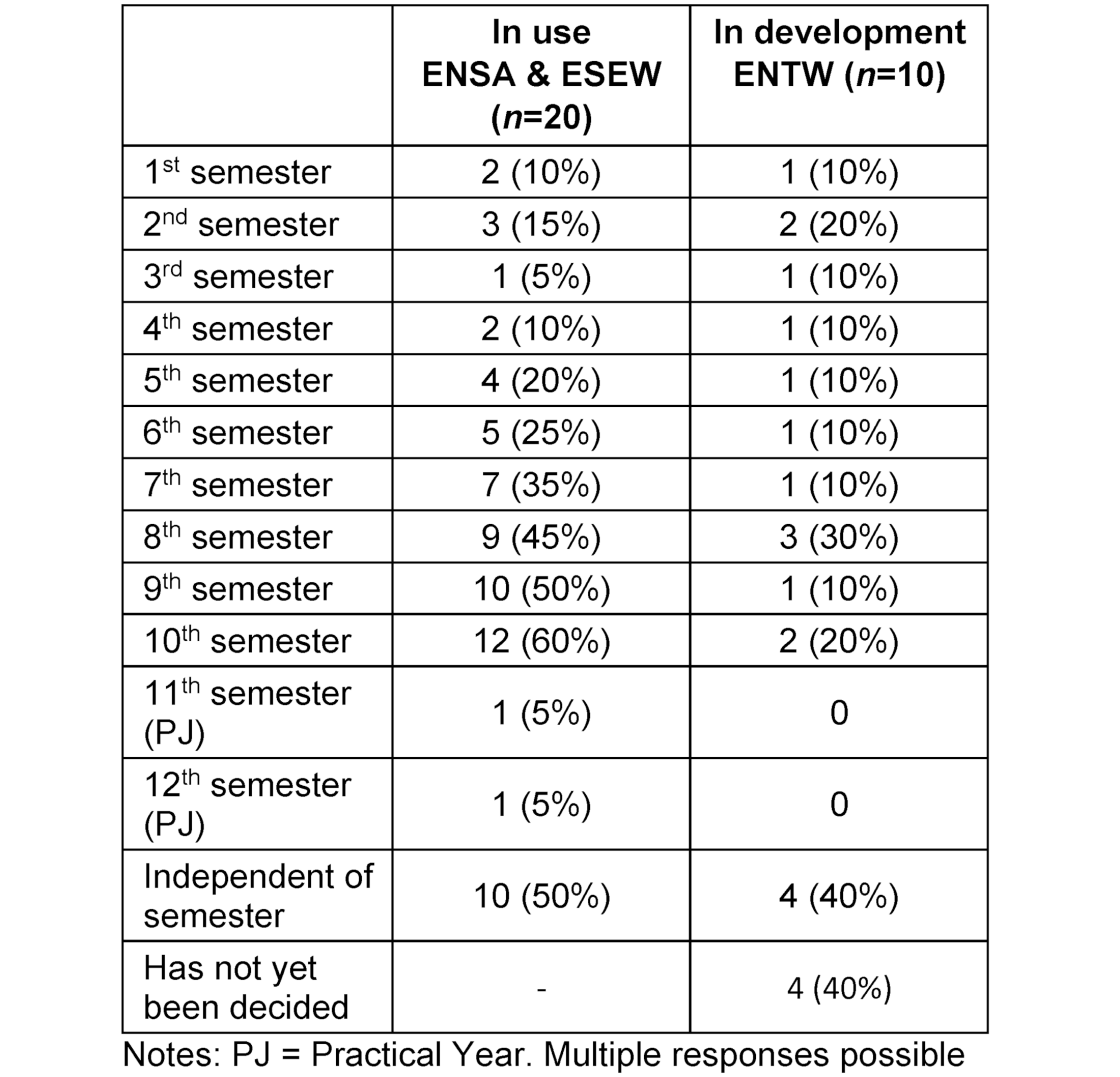
Semesters in which VR is already used in teaching or currently in development

**Table 9:**
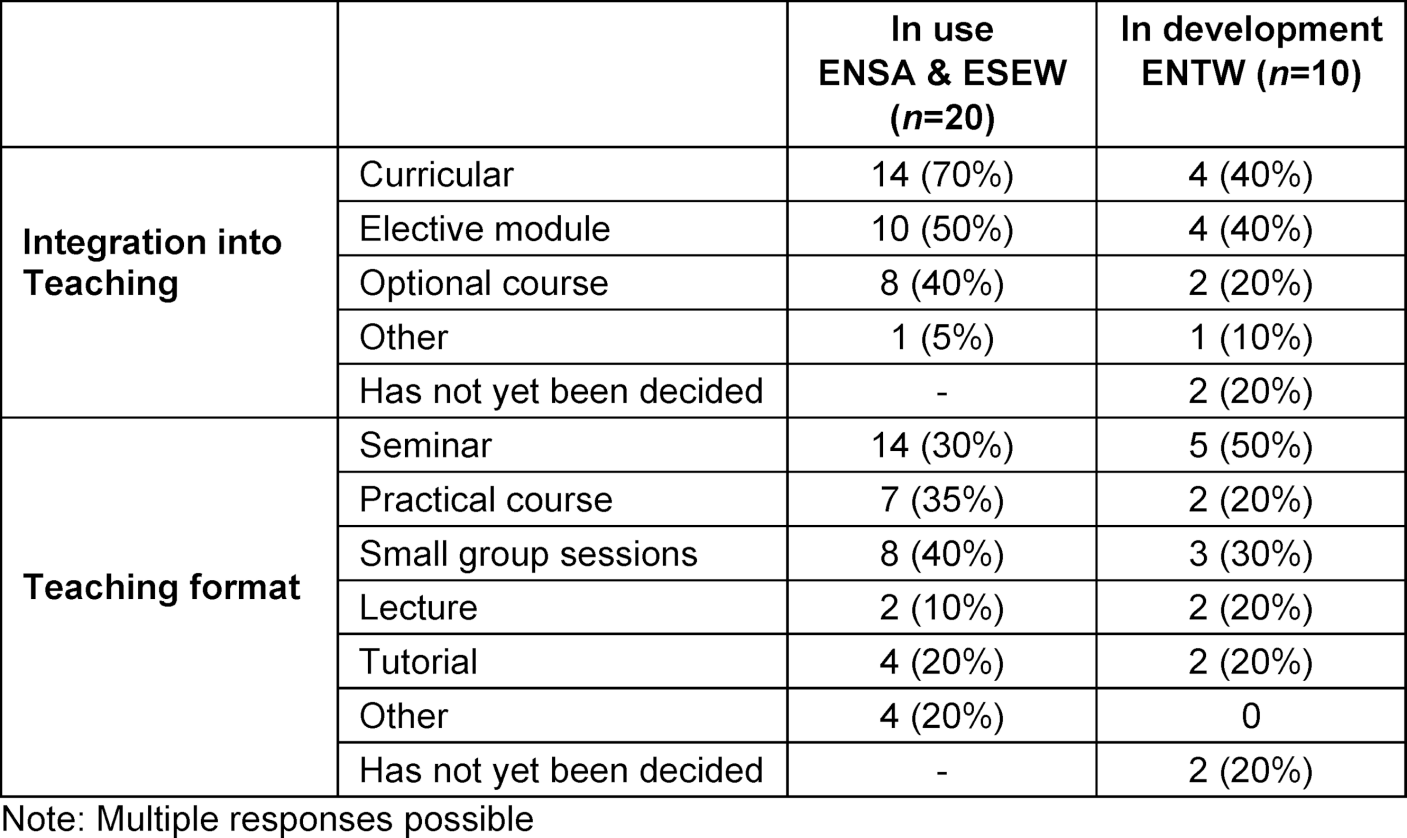
Integration into teaching and course formats

**Table 10:**
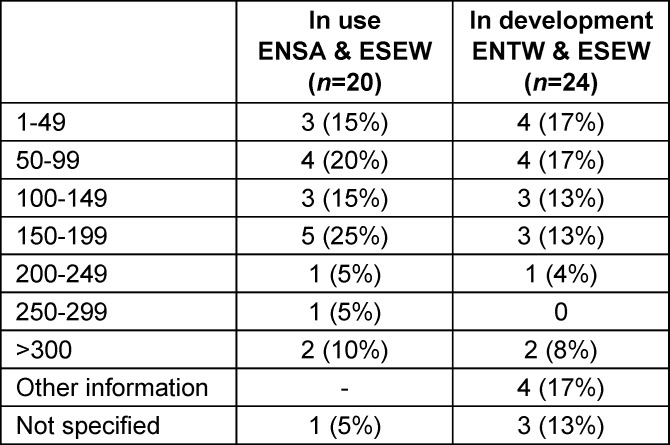
Participants per semester at locations with existing use of VR in teaching and at locations with development of courses with VR components

**Table 11:**
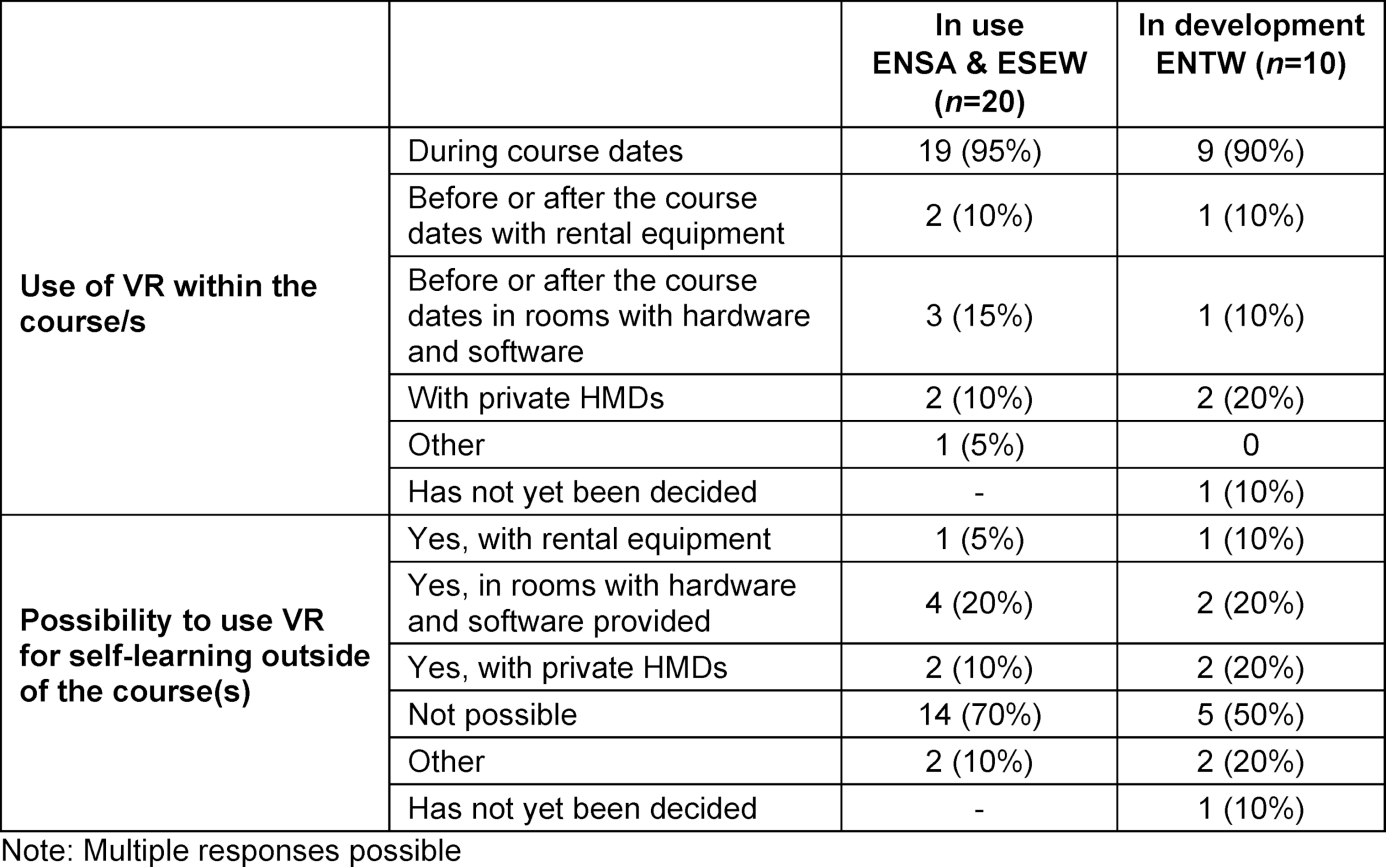
Didactic implementation of the use of VR through synchronous/asynchronous teaching

**Table 12:**
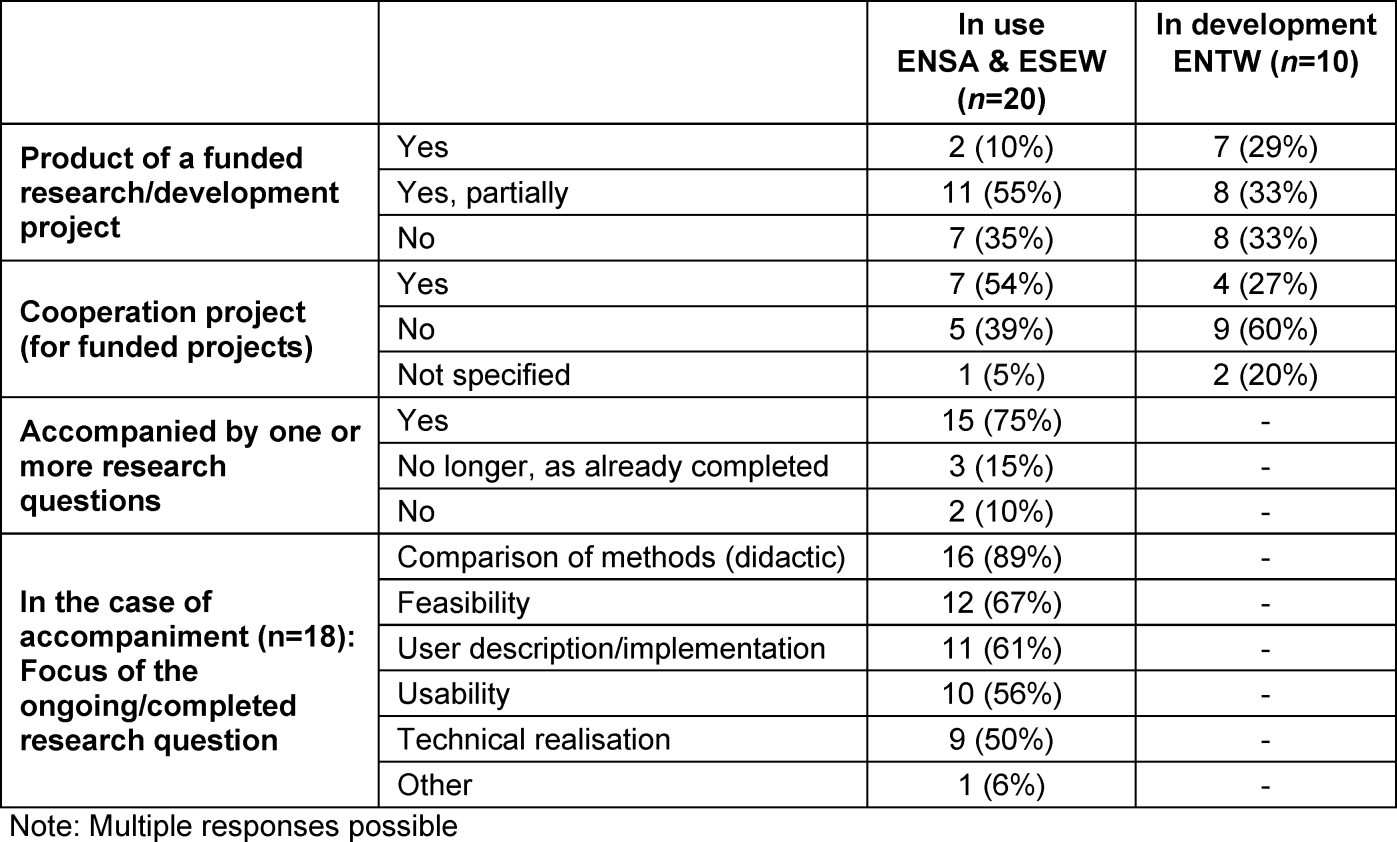
Integration of VR in research projects or teaching research

**Table 13:**
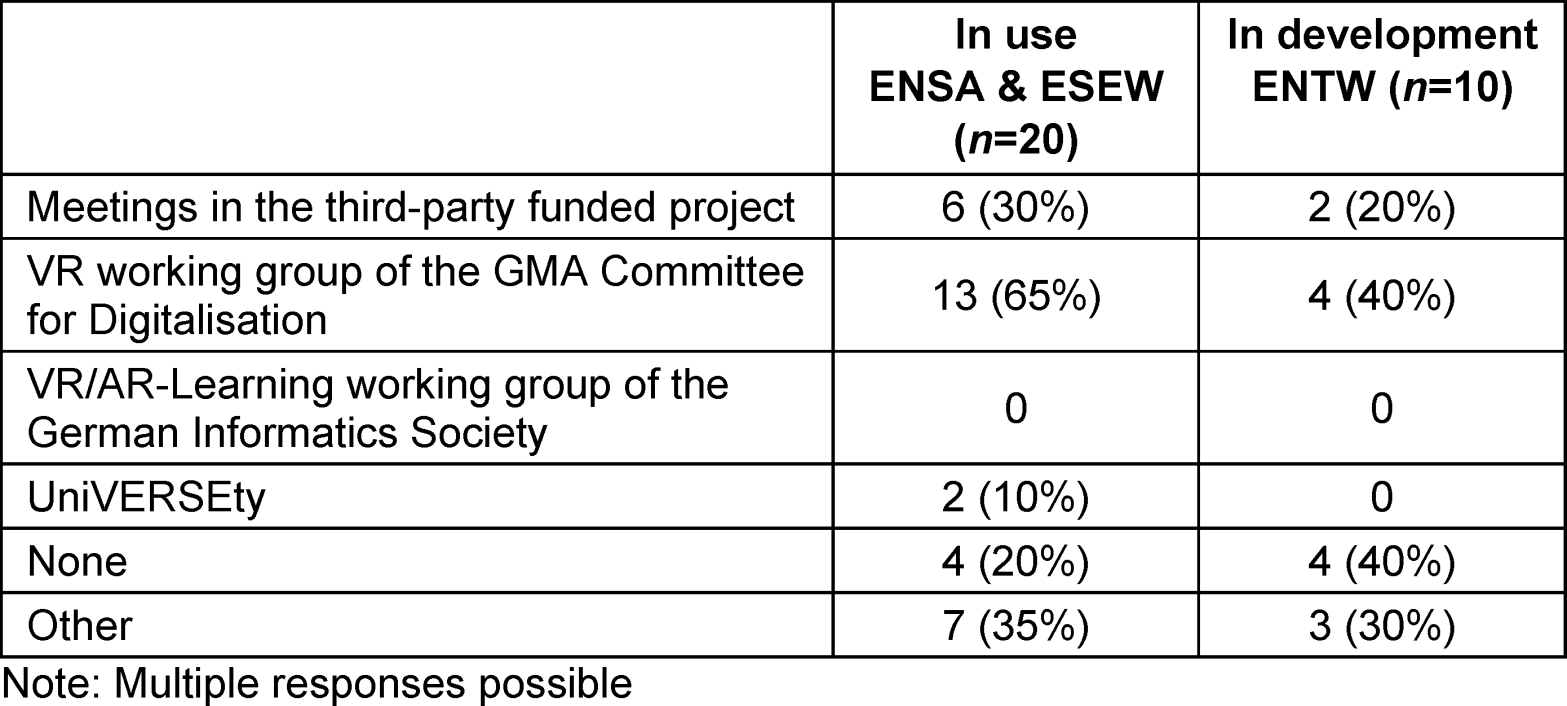
Utilisation of cross-location exchange forums on VR in teaching
