# Supplementary material for: Current use of virtual reality in medical education in Germany, Austria, and Switzerland: Results of an online survey among medical faculties
Source: GMS J Med Educ. 2025 Nov 17;42(5):Doc61. doi: 10.3205/zma001785 (PMC12661504; doi:10.3205/zma001785)
Supplement: Questionnaire (only in German) [file JME-42-61-s-001.pdf]

Attachment 1: Questionnaire (only in German)

Attachment 1 to Willemer MC, Meyerheim M, Mergen M, Schulze H, Eiler TJ, Mayer L, Romeike B, Hätscher O, Speidel R, Junga A. *Current use of virtual reality in medical education in Germany, Austria, and Switzerland: Results of an online survey among medical faculties.* GMS J Med Educ. 2025;42(5):Doc61. DOI: 10.3205/zma001785

Fragebogen

1 Startseite

"Status Quo von Virtual Reality in der medizinischen Ausbildung der DACH-Region"

Wir laden Sie herzlich dazu ein, an der folgenden Online-Befragung teilzunehmen, wenn Sie für die digitale Lehre in der human- oder zahnmedizinischen Ausbildung zuständig sind (z.B. Abteilungsleiter:in eines Dekanats in der DACH-Region) oder Sie VR bereits für Lehrzwecke einsetzen oder entwickeln (z.B. Dozent:in).

Die etwa 15-minütige Befragung wird von der Universität Ulm durchgeführt und erfasst Meinungen und Informationen rund um den didaktischen Einsatz von VR. Das Ziel der Erhebung ist es, den Status Quo von Virtual Reality in der medizinischen Ausbildung der DACH-Region zu erfassen und in einer Überblicksarbeit zu publizieren. Dadurch möchten wir unter anderem die Vernetzung zwischen VR-Anwendern stärken, um Kooperationen zu ermöglichen und Mehrfachentwicklungen zu vermeiden.

Die Ergebnisse werden standortspezifisch ausgewertet. In der angestrebten Publikation werden Ihre Angaben somit ohne Namen und ohne Kontaktdaten unter Ihrer Einrichtung geführt. Dadurch könnte ein indirekter Rückschluss auf Ihre Person möglich sein. Um an der Befragung teilnehmen zu können, ist die Verarbeitung Ihrer personenbezogenen Daten erforderlich.

Widerrufsbelehrung

Diese Einwilligung i. S. d. Art. 6 Abs. 1 lit. a) i. V. m. Art. 7 DSGVO kann jederzeit ohne Angabe von Gründen schriftlich widerrufen werden. Der Widerruf ist per E-Mail an „Robert.Speidel@uni-ulm.de“ zu richten. Die Rechtmäßigkeit der bis zum Widerruf erfolgten Datenverarbeitung bleibt vom Widerruf unberührt. Soweit die Einwilligung nicht widerrufen wird, gilt sie zeitlich unbeschränkt. Sie erfolgt freiwillig; aus der Verweigerung der Einwilligung oder ihrem Widerruf entstehen keine Nachteile. Weitere Informationen zur Datenverarbeitung finden Sie in der Datenschutzerklärung.

Für Fragen und Feedback wenden Sie sich bitte an:

Robert Speidel
Medizinische Fakultät Ulm
Kompetenzzentrum eEducation in der Medizin BW
Robert.Speidel@uni-ulm.de
+49 (0)731 50 33685

Einverständniserklärung

Einsicht und Download der Datenschutzerklärung

"Ich erkläre freiwillig, dass ich die Datenschutzerklärung zur Kenntnis genommen habe und mit der darin beschriebenen Verarbeitung meiner personenbezogenen Daten einverstanden bin. Ich bin über den Umfang und Zweck der Datenerhebung und Datenverarbeitung sowie über mein Widerrufsrecht informiert worden. Eine Kopie der Datenschutzerklärung konnte ich herunterladen und speichern."

- ◯ Ja
- ◯ Nein

2.1 Endseite - Keine Einwilligung

Sie haben der Datenverarbeitung nicht zugestimmt und können daher nicht an der Befragung teilnehmen.

Sollten Sie Ihre Meinung ändern, leeren Sie bitte den Cache und löschen Sie die Cookies Ihres Browsers, bevor Sie die Befragung erneut über den folgenden Link aufrufen: https://ww2.unipark.de/uc/VR-Status

Für Fragen oder Feedback wenden Sie sich bitte an:

Robert Speidel
Medizinische Fakultät Ulm
Kompetenzzentrum eEducation in der Medizin BW
Robert.Speidel@uni-ulm.de
+49 (0)731 50 33685

3 Standort

In welcher Einrichtung sind Sie tätig und wo befindet sich diese?

z.B. Dekanat der Medizinische Fakultät Ulm

Einrichtung ____________ Stadt ____________

Die folgenden drei Fragen dienen nur zur Validierung Ihrer Angaben und für mögliche Rückfragen.

Wie heißen Sie?

Ihr Name wird nicht publiziert.

Vorname ____________ Nachname ____________

In welcher Position sind Sie an Ihrer Einrichtung tätig?

Ihre Position wird nicht publiziert.

____

Unter welcher Telefonnummer und/oder E-Mail-Adresse dürfen wir Sie kontaktieren?

Ihre Kontaktdaten werden nicht publiziert.

Telefonnummer ____________ E-Mail ____________

4 Ursprung (I)

Wird VR bereits an Ihrer Einrichtung für die Lehre eingesetzt und/oder entwickeln Sie derzeit eine Lehrveranstaltung mit VR-Anteilen? ?

VR-Brillen, die Smartphones als Displays verwenden, sind nicht gemeint!

☐ Ja, bestehender Einsatz von VR in der Lehre

☐ Ja, aktuelle Entwicklung von Lehrveranstaltungen mit VR-Anteilen

☐ Nein

4.1.1 Non-User (I)

Beabsichtigen Sie an Ihrer Einrichtung VR zukünftig in der Lehre einzusetzen?

○ Ja

○ Nein

4.1.2.1 Non-User (II)

Nach dieser Seite gelangen Sie zum Ende des Fragebogens und können nicht mehr zurück.

Gibt es bestimmte Gründe, warum Sie an Ihrer Einrichtung nicht beabsichtigen, VR zukünftig in der Lehre einzusetzen? Falls ja, welche?

4.1.2.1.1 Endseite nach future

Vielen Dank für Ihre Teilnahme an der Befragung!

Haben Sie noch Fragen oder Feedback? Dann wenden Sie sich bitte an:

Robert Speidel
Medizinische Fakultät Ulm
Kompetenzzentrum eEducation in der Medizin BW
Robert.Speidel@uni-ulm.de
+49 (0)731 50 33685

4.1.3.1 Non-User (III)

Nach dieser Seite gelangen Sie zum Ende des Fragebogens und können nicht mehr zurück.

Für welche/n medizinischen Fachbereich/e beabsichtigen Sie, VR zukünftig für die Lehre einzusetzen? ?

Vorklinischer Bereich	Klinischer Bereich			Sonstiges
☐ Makroskopische Anatomie	☐ Pathologie	☐ Neurologie	☐ Hygiene, Mikrobiologie, Virologie	☐ Der Bereich steht noch nicht fest.
☐ Mikroskopische Anatomie	☐ Notfallmedizin	☐ Neurochirurgie	☐ Infektiologie, Immunologie	☐ sonstiger Bereich:
☐ Physiologie	☐ Allgemeinmedizin	☐ Dermatologie	☐ Klinische Chemie	
☐ Biologie	☐ Innere Medizin	☐ Orthopädie	☐ Pharmakologie	
☐ Biochemie	☐ Anästhesiologie	☐ HNO	☐ Arbeitsmedizin / Sozialmedizin	
☐ Chemie	☐ Chirurgie	☐ Radiologie	☐ Rechtsmedizin	
☐ Physik	☐ Gynäkologie	☐ Pädiatrie	☐ Klinische Umweltmedizin	
☐ Psychologie / Soziologie	☐ Urologie	☐ Palliativ-/ Schmerzmedizin	☐ Geriatrie	
☐ Terminologie	☐ Psychiatrie u. Psychotherapie	☐ Ophthalmologie	☐ Prävention	
	☐ Psychosomatische Medizin	☐ Humangenetik		

Aus welchem Grund beabsichtigen Sie den zukünftigen Einsatz von VR für die Lehre? ?

- ☐ Verbesserung/Weiterentwicklung der bestehenden Lehre
- ☐ Stärkung des Praxisbezugs in der Ausbildung
- ☐ Erschließung neuer Lerninhalte
- ☐ Vorbereitung der Studierenden auf den klinischen Einsatz von VR
- ☐ Steigerung der Attraktivität des Standorts für Studierende
- ☐ Erfüllung von gesetzlichen Rahmenbedingungen (z.B. NKLM)
- ☐ Sonstiges

4.1.3.1.1 Endseite nach future

Vielen Dank für Ihre Teilnahme an der Befragung!

Haben Sie noch Fragen oder Feedback? Dann wenden Sie sich bitte an:

Robert Speidel
Medizinische Fakultät Ulm
Kompetenzzentrum eEducation in der Medizin BW
Robert.Speidel@uni-ulm.de
+49 (0)731 50 33685

4.2.1 Ursprung (IIa)

Die folgenden Fragen beziehen sich auf Ihren bereits bestehenden Einsatz von VR in der Lehre.

Seit wann setzen Sie VR in der Lehre ein?

Nur Monat und Jahr sind von Belang. Der genaue Tag wird nicht ausgewertet.

Welche Ziele verfolgen Sie bei Ihrem bestehenden Einsatz von VR in der Lehre? ?

- ☐ Verbesserung/Weiterentwicklung der bestehenden Lehre
- ☐ Stärkung des Praxisbezugs in der Ausbildung
- ☐ Erschließung neuer Lerninhalte
- ☐ Vorbereitung der Studierenden auf den klinischen Einsatz von VR
- ☐ Steigerung der Attraktivität des Standorts für Studierende
- ☐ Erfüllung von gesetzlichen Rahmenbedingungen (z.B. NKLM)
- ☐ Sonstiges

Was hat Sie dazu bewogen, VR in der Lehre einzusetzen? ?

- ☐ Persönliches Interesse an der Technologie
- ☐ Didaktisches Potenzial von VR
- ☐ Vorgabe der/des Vorgesetzten
- ☐ Projektausschreibung
- ☐ Forderung durch gesetzliche Rahmenbedingungen (z.B. NKLM)
- ☐ Sonstiges

4.2.2 Ursprung (IIIa)

Wie haben Sie die Beschaffung der Hardware und die Lizenzierung/Entwicklung der Software finanziert? ?

z. B. VR-Brillen und Computer

- ☐ Drittmittel
- ☐ Haushalt der Medizinischen Fakultät/Universität
- ☐ Haushalt des Universitätsklinikums
- ☐ Haushalt externer Kooperations:partnerinnen
- ☐ Landesmittel für Lehre (z.B. Sonderlinie Medizin)
- ☐ Sonstige, und zwar: ________

Wie erfolgt die Finanzierung der laufenden Kosten von VR in der Lehre? ?

z. B. Finanzierung für Erneuerung von Lizenzen, Instandhaltung von Hardware und Personalkosten.

- ☐ Drittmittel
- ☐ Haushalt der Medizinischen Fakultät/Universität
- ☐ Haushalt des Universitätsklinikums
- ☐ Haushalt externer Kooperationspartner:innen
- ☐ Landesmittel für Lehre (z.B. Sonderlinie Medizin)
- ☐ Sonstige, und zwar: ________

4.2.2.1.1 Ursprung (IVa)

Wer stellt/e Ihnen die Drittmittel zur Verfügung?

- ○ ________
- ○ Keine Angabe

Endet der Einsatz von VR nach der Projektförderung?

- ◯ Ja
- ◯ Zum Teil
- ◯ Nein
- ◯ Das steht noch nicht fest.

4.2.3 Technische Umsetzung (Ia)

Welche VR-Brillen haben Sie in Ihrer Einrichtung für die Lehre?

?

HTC Vive	HP	Oculus	Meta Quest	Pico	Samsung	Valve Index	Varjo	Sonstige
☐ HTC Vive Cosmos	☐ HP Reverb	☐ Oculus Go	☐ Meta Quest	☐ Pico Neo CV	☐ Samsung Odyssey	☐ Valve Index	☐ Varjo VR-1	☐ ________
☐ HTC Vive Cosmos Elite	☐ HP Reverb G2	☐ Oculus Rift	☐ Meta Quest 2	☐ Pico G2 4K	☐ Samsung Odyssey+		☐ Varjo VR-2	
☐ HTC Vive Focus		☐ Oculus Rift S	☐ Meta Quest Pro	☐ Pico Neo 3 Link			☐ Varjo VR-3	
☐ HTC Vive Focus Plus				☐ Pico Neo 3 Pro			☐ Varjo Aero	
☐ HTC Vive Focus 3				☐ Pico Neo 3 Pro Eye			☐ Varjo XR-3	
☐ HTC Vive				☐ Pico 4				
☐ HTC Vive Pro								
☐ HTC Vive Pro 2								
☐ HTC Vive Pro Eye								
☐ HTC Vive XR Elite								

Wie viele VR-Brillen haben Sie in Ihrer Einrichtung für die Lehre?

- ◯ 1-4
- ◯ 5-15
- ◯ 16-30
- ◯ > 30

Nutzen Sie außer Controllern/Hand-Tracking noch ergänzende Ein- und Ausgabegeräte?

z.B. Datenhandschuhe, nachgebildete Geräte oder Bodysuits

- ◯ Ja, und zwar: ____________
- ◯ Nein

4.2.4 Technische Umsetzung (IIa1)

Verwenden Sie für den Einsatz von VR in der Lehre dezidierte Räumlichkeiten?

- ◯ Ja, es werden dezidierte Räumlichkeiten genutzt.
- ◯ Nein, es werden beliebige Räumlichkeiten genutzt.
- ◯ Es werden sowohl dezidierte als auch beliebige Räumlichkeiten genutzt.

Verwenden Sie VR-Software mit einem Multiplayer-Modus, sodass sie von mehreren Studierenden zusammen genutzt werden kann?

- ◯ Ja
- ◯ Nein

Verwenden Sie VR-Software, die von Studierenden alleine (ohne Betreuung) verwendet werden kann? ?

- ☐ Ja, eine Einweisung durch einen Betreuenden ist allerdings notwendig.
- ☐ Ja, eine Einweisung durch einen Betreuenden ist nicht notwendig.
- ☐ Nein

4.2.5 Technische Umsetzung (IIa2)

Verwenden Sie VR-Software, die von Studierenden auch von zu Hause aus genutzt werden kann?

?

- ☐ Ja, die VR-Software setzt jedoch eine VR-Brille voraus.
  (VR-Brillen, die Smartphones als Displays verwenden, sind nicht gemeint)
- ☐ Ja, die VR-Software kann mit VR-Brillen genutzt werden, die Smartphones als Displays verwenden.
  (z.B. Google Cardboard oder Samsung Gear VR)
- ☐ Ja, die VR-Software kann mit nicht-immersiven Geräten genutzt werden.
  (PC/Mac, Laptop, Tablet oder Smartphone)
- ☐ Nein

Wer ist für die Wartung und die Netzwerkintegration der Hardware zuständig?

?

- ☐ IT-Mitarbeitende der Medizinischen Fakultät/Universität
- ☐ IT-Mitarbeitende der Klinik
- ☐ Externe Dienstleister
- ☐ Sonstige: __________

Nutzen Sie in Ihrer Lehre VR-Software, die nicht von oder mit Ihrer Einrichtung entwickelt wurde?

VR-kompatible Lernpakete, die mit 360°-Medien erstellt wurden, sind nicht gemeint.

- ☐ Ja, mit Nutzungsrechten einer kommerziellen Lizenz
- ☐ Ja, mit Nutzungsrechten einer CC-Lizenz
- ☐ Nein
- ☐ Sonstiges __________

Hat Ihre Einrichtung VR-Software, die bereits in Ihrer Lehre eingesetzt wird, entwickelt oder mitentwickelt?

?

VR-kompatible Lernpakete, die mit 360°-Medien erstellt wurden, sind nicht gemeint.

- ☐ Ja, eigenständige VR-Software Entwicklung (inkl. 3D-Modellierung und Programmierung)
- ☐ Ja, Beteiligung mit medizinischem Fachwissen
- ☐ Ja, Beteiligung mit didaktischem Fachwissen
- ☐ Nein

4.2.5.1.1 Technische Umsetzung (IIIa)

Stellen Sie Ihre VR-Software anderen Nutzer:innen zur Verfügung oder ist dies zukünftig geplant?

?

- ☐ Ja, kommerziell
- ☐ Ja, kostenlos (z.B. unter einer CC-Lizenz)
- ☐ Nein
- ☐ Das steht noch nicht fest.

Mit welchen Einrichtungen und/oder Unternehmen haben Sie bei der Entwicklung der VR-Software zusammengearbeitet?

4.2.6 Didaktische Umsetzung (la)

Für welche/n medizinischen Fachbereich/e setzen Sie VR in der Lehre ein?

?

Vorklinischer Bereich

- ☐ Makroskopische Anatomie
- ☐ Mikroskopische Anatomie
- ☐ Physiologie
- ☐ Biologie
- ☐ Biochemie
- ☐ Chemie
- ☐ Physik
- ☐ Psychologie / Soziologie
- ☐ Terminologie

Klinischer Bereich

- ☐ Pathologie
- ☐ Notfallmedizin
- ☐ Allgemeinmedizin
- ☐ Innere Medizin
- ☐ Anästhesiologie
- ☐ Chirurgie
- ☐ Gynäkologie
- ☐ Urologie
- ☐ Psychiatrie u. Psychotherapie
- ☐ Psychosomatische Medizin
- ☐ Neurologie
- ☐ Neurochirurgie
- ☐ Dermatologie
- ☐ Orthopädie
- ☐ HNO
- ☐ Radiologie
- ☐ Pädiatrie
- ☐ Palliativ-/ Schmerzmedizin
- ☐ Ophthalmologie
- ☐ Humangenetik
- ☐ Hygiene, Mikrobiologie, Virologie
- ☐ Infektiologie, Immunologie
- ☐ Klinische Chemie
- ☐ Pharmakologie
- ☐ Arbeitsmedizin / Sozialmedizin
- ☐ Rechtsmedizin
- ☐ Klinische Umweltmedizin
- ☐ Geriatrie
- ☐ Prävention

Sonstige Bereiche

Wie binden Sie VR aktuell in der Lehre ein? ?

- [ ] Curricular
- [ ] Wahlfach
- [ ] Fakultativer Kurs
- [ ] Sonstiges: ____

4.2.7 Didaktische Umsetzung (IIa)

In welchen Lehrformaten verwenden Sie VR? ?

- [ ] Seminar
- [ ] Praktikum
- [ ] Kleingruppenunterricht
- [ ] Vorlesung
- [ ] Tutorium
- [ ] Sonstiges: ____

In welchen Semestern verwenden Sie VR in der Lehre? ?

[ ] 1. Semester	[ ] 5. Semester	[ ] 9. Semester	[ ] semesterunabhängig
[ ] 2. Semester	[ ] 6. Semester	[ ] 10. Semester	
[ ] 3. Semester	[ ] 7. Semester	[ ] 11. Semester (PJ)	
[ ] 4. Semester	[ ] 8. Semester	[ ] 12. Semester (PJ)	

Wie viele Studierende nutzen VR aktuell pro Semester an Ihrer Einrichtung? (circa)

Bitte geben Sie eine Zahl ein.

____

4.2.8 Didaktische Umsetzung (IIIa)

Wie ist der Einsatz von VR innerhalb der Lehrveranstaltung/en organisiert?

?

- [ ] Die Studierenden nutzen VR während der Kurstermine.
- [ ] Die Studierenden nutzen VR vor oder nach den Kursterminen eigenständig mit Leihgeräten.
- [ ] Die Studierenden nutzen VR vor oder nach den Kursterminen in Räumlichkeiten, in denen Hard- und Software bereitgestellt wird.
- [ ] Die Studierenden nutzen VR auf privaten VR-Brillen.
- [ ] Sonstiges:

Können Studierende VR auch außerhalb der Lehrveranstaltung/en für das selbstbestimmte Lernen nutzen?

?

- [ ] Ja, mit Leihgeräten
- [ ] Ja, in Räumlichkeiten unserer Einrichtung, in denen Hard- und Software bereitgestellt wird
- [ ] Ja, mit privaten VR-Brillen
- [ ] Nein
- [ ] Sonstiges:

4.2.9 Kooperation (Ia)

Ist Ihr bestehender Einsatz von VR in der Lehre Teil oder Produkt eines geförderten Forschungs- und/oder Entwicklungsprojekts?

- ◯ Ja
- ◯ Ja, zum Teil
- ◯ Nein

4.2.9.1.1 Kooperation (II)

Wie heißt oder hieß Ihr gefördertes VR-Projekt bzw. Ihre geförderten VR-Projekte?

?

Falls eine Projektwebsite vorhanden ist, bitte Link angeben.

Handelt/e es sich bei dem geförderten VR-Projekt bzw. den geförderten VR-Projekten um Kooperationsvorhaben?
Falls ja, wer ist/war an dem/den Vorhaben beteiligt?

- ◯ Ja, Beteiligte: ____
- ◯ Nein

4.2.10 Forschung (I)

Begleiten Sie den Einsatz von VR in der Lehre mit einer oder mehreren wissenschaftlichen Fragestellung/en?

- ◯ Ja
- ◯ Nicht mehr, die wissenschaftliche Untersuchung ist bereits abgeschlossen.
- ◯ Nein

4.2.10.1.1 Forschung (II)

Welchen Schwerpunkt hat/haben bzw. hatte/hatten die wissenschaftliche/n Fragestellung/en? ?

- ☐ Technische Umsetzung
- ☐ Usability
- ☐ Methodenvergleich (didaktisch)
- ☐ Anwenderbeschreibung / Implementierung
- ☐ Machbarkeit
- ☐ Sonstiges / Kommentar: ____

4.3.1.1 Ursprung IIb

Welche Ziele verfolgen Sie bei Ihrem geplanten Einsatz von VR in der Lehre? ?

- ☐ Verbesserung/Weiterentwicklung der bestehenden Lehre
- ☐ Stärkung des Praxisbezugs in der Ausbildung
- ☐ Erschließung neuer Lerninhalte
- ☐ Vorbereitung der Studierenden auf den klinischen Einsatz von VR
- ☐ Steigerung der Attraktivität des Standorts für Studierende
- ☐ Erfüllung von gesetzlichen Rahmenbedingungen (z.B. NKLM)
- ☐ Sonstiges

Was hat Sie dazu bewogen, VR in der Lehre einsetzen zu wollen? ?

- ☐ Persönliches Interesse an der Technologie
- ☐ Didaktisches Potenzial von VR
- ☐ Vorgabe der/des Vorgesetzten
- ☐ Projektausschreibung
- ☐ Forderung durch gesetzliche Rahmenbedingungen (z.B. NKLM)
- ☐ Sonstiges

4.3.1.2 Ursprung (IIIb)

Wie haben Sie die Beschaffung der Hardware und die Lizenzierung/Entwicklung der Software finanziert bzw. wie planen Sie dies? ?

z. B. VR-Brillen und Computer

- ☐ Drittmittel
- ☐ Haushalt der Medizinischen Fakultät/Universität
- ☐ Haushalt des Universitätsklinikums
- ☐ Haushalt externer Kooperations:partnerinnen
- ☐ Landesmittel für Lehre (z.B. Sonderlinie Medizin)
- ☐ Sonstige, und zwar:
- ☐ Das steht noch nicht fest.

Wie werden die laufenden Kosten des Einsatzes von VR in der Lehre zukünftig an Ihrer Einrichtung finanziert?

?

z. B. Finanzierung für Erneuerung von Lizenzen, Instandhaltung von Hardware und Personalkosten.

- ☐ Drittmittel
- ☐ Haushalt der Medizinischen Fakultät/Universität
- ☐ Haushalt des Universitätsklinikums
- ☐ Haushalt externer Kooperationspartner:innen
- ☐ Landesmittel für Lehre (z.B. Sonderlinie Medizin)
- ☐ Sonstige, und zwar: ______
- ☐ Das steht noch nicht fest.

4.3.1.2.1.1 Ursprung (IVb)

Wer stellt Ihnen die Drittmittel zur Verfügung?

- ○ ______
- ○ Keine Angabe

Endet der Einsatz von VR nach der Projektförderung?

- ○ Ja
- ○ Zum Teil
- ○ Nein
- ○ Das steht noch nicht fest.

4.3.1.3 Technische Umsetzung (Ib)

Welche VR-Brillen werden Sie verwenden?

?

HTC Vive

- [ ] HTC Vive Cosmos
- [ ] HTC Vive Cosmos Elite
- [ ] HTC Vive Focus
- [ ] HTC Vive Focus Plus
- [ ] HTC Vive Focus 3
- [ ] HTC Vive
- [ ] HTC Vive Pro
- [ ] HTC Vive Pro 2
- [ ] HTC Vive Pro Eye
- [ ] HTC Vive XR Elite

HP

- [ ] HP Reverb
- [ ] HP Reverb G2

Oculus

- [ ] Oculus Go
- [ ] Oculus Rift
- [ ] Oculus Rift S

Meta Quest

- [ ] Meta Quest
- [ ] Meta Quest 2
- [ ] Meta Quest Pro

Pico

- [ ] Pico Neo CV
- [ ] Pico G2 4K
- [ ] Pico Neo 3 Link
- [ ] Pico Neo 3 Pro
- [ ] Pico Neo 3 Pro Eye
- [ ] Pico 4

Samsung

- [ ] Samsung Odyssey
- [ ] Samsung Odyssey+

Valve Index

- [ ] Valve Index

Varjo

- [ ] Varjo VR-1
- [ ] Varjo VR-2
- [ ] Varjo VR-3
- [ ] Varjo Aero
- [ ] Varjo XR-3

Sonstige

- [ ] Das steht noch nicht fest.
- [ ] ______

Wie viele VR-Brillen werden Sie verwenden?

- ○ 1-4
- ○ 5-15
- ○ 16-30
- ○ > 30
- ○ Das steht noch nicht fest.

Planen Sie außer Controllern/Hand-Tracking noch ergänzende Ein- und Ausgabegeräte zu nutzen?

z.B. Datenhandschuhe, nachgebildete Geräte oder Bodysuits

- ○ Ja, und zwar: ______
- ○ Nein
- ○ Das steht noch nicht fest.

4.3.1.4 Technische Umsetzung (IIb)

Werden Sie für den Einsatz von VR in der Lehre dezidierte Räumlichkeiten nutzen?

- ○ Ja, es werden dezidierte Räumlichkeiten genutzt.
- ○ Nein, es werden beliebige Räumlichkeiten genutzt.
- ○ Es werden sowohl dezidierte als auch beliebige Räumlichkeiten genutzt.
- ○ Das steht noch nicht fest.

Werden Sie VR-Software mit einem Multiplayer-Modus verwenden, sodass sie von mehreren Studierenden zusammen genutzt werden kann?

- ○ Ja
- ○ Nein
- ○ Das steht noch nicht fest.

Werden Sie VR-Software verwenden, die von Studierenden alleine (ohne Betreuung) genutzt werden kann? ?

- ☐ Ja, eine Einweisung durch einen Betreuenden ist allerdings notwendig.
- ☐ Ja, eine Einweisung durch einen Betreuenden ist nicht notwendig.
- ☐ Nein
- ☐ Das steht noch nicht fest.

Werden Sie VR-Software verwenden, die von Studierenden auch von zu Hause aus genutzt werden kann? ?

- [ ] Ja, die VR-Software setzt jedoch eine VR-Brille voraus.

  (VR-Brillen, die Smartphones als Displays verwenden, sind nicht gemeint)
- [ ] Ja, die VR-Software kann mit VR-Brillen genutzt werden, die Smartphones als Displays verwenden.

  (z.B. Google Cardboard oder Samsung Gear VR)
- [ ] Ja, die VR-Software kann mit nicht-immersiven Geräten genutzt werden.

  (PC/Mac, Laptop, Tablet oder Smartphone)
- [ ] Nein
- [ ] Das steht noch nicht fest.

Wer ist für die Wartung und die Netzwerkintegration der Hardware vorgesehen? ?

- [ ] IT-Mitarbeitende der Medizinischen Fakultät/Universität
- [ ] IT-Mitarbeitende der Klinik
- [ ] Externe Dienstleister
- [ ] Sonstige: ____________________
- [ ] Das steht noch nicht fest.

4.3.1.5 Didaktische Umsetzung (Ib)

Für welche/n medizinischen Fachbereich/e entwickeln Sie derzeit Ihre Lehrveranstaltung/en mit VR-Anteilen? ?

Vorklinischer Bereich

- ☐ Makroskopische Anatomie
- ☐ Mikroskopische Anatomie
- ☐ Physiologie
- ☐ Biologie
- ☐ Biochemie
- ☐ Chemie
- ☐ Physik
- ☐ Psychologie / Soziologie
- ☐ Terminologie

Klinischer Bereich

- ☐ Pathologie
- ☐ Notfallmedizin
- ☐ Allgemeinmedizin
- ☐ Innere Medizin
- ☐ Anästhesiologie
- ☐ Chirurgie
- ☐ Gynäkologie
- ☐ Urologie
- ☐ Psychiatrie u. Psychotherapie
- ☐ Psychosomatische Medizin
- ☐ Neurologie
- ☐ Neurochirurgie
- ☐ Dermatologie
- ☐ Orthopädie
- ☐ HNO
- ☐ Radiologie
- ☐ Pädiatrie
- ☐ Palliativ-/ Schmerzmedizin
- ☐ Ophthalmologie
- ☐ Humangenetik
- ☐ Hygiene, Mikrobiologie, Virologie
- ☐ Infektiologie, Immunologie
- ☐ Klinische Chemie
- ☐ Pharmakologie
- ☐ Arbeitsmedizin / Sozialmedizin
- ☐ Rechtsmedizin
- ☐ Klinische Umweltmedizin
- ☐ Geriatrie
- — Prävention

Sonstige Bereiche

- ☐ Das steht noch nicht fest.
- ☐ sonstiger Bereich: ____

Wie werden Sie Ihre Lehrveranstaltung/en mit VR-Anteilen in die Lehre einbinden? ?

- ☐ Curricular
- ☐ Wahlfach
- ☐ Fakultativer Kurs
- ☐ Sonstiges: ____
- ☐ Das steht noch nicht fest.

4.3.1.6 Didaktische Umsetzung (IIb)

In welchem Lehrformat werden Sie Ihre Lehrveranstaltung/en mit VR-Anteilen anbieten? ?

- [ ] Seminar
- [ ] Praktikum
- [ ] Kleingruppenunterricht
- [ ] Vorlesung
- [ ] Tutorium
- [ ] Sonstiges:
- [ ] Das steht noch nicht fest.

In welchem/n Semester/n wird/werden Ihre Lehrveranstaltung/en mit VR-Anteilen stattfinden? ?

☐ 1. Semester	☐ 5. Semester	☐ 9. Semester	☐ semesterunabhängig
☐ 2. Semester	☐ 6. Semester	☐ 10. Semester	☐ Das steht noch nicht fest.
☐ 3. Semester	☐ 7. Semester	☐ 11. Semester (PJ)	
☐ 4. Semester	☐ 8. Semester	☐ 12. Semester (PJ)	

4.3.1.7 Didaktische Umsetzung (IIIb)

Wie ist der Einsatz von VR <u>innerhalb</u> der sich in Entwicklung befindlichen Lehrveranstaltung/en geplant? ?

- [ ] Die Studierenden nutzen VR während der Kurstermine.
- [ ] Die Studierenden nutzen VR vor oder nach den Kursterminen eigenständig mit Leihgeräten.
- [ ] Die Studierenden nutzen VR vor oder nach den Kursterminen in Räumlichkeiten, in denen Hard- und Software bereitgestellt wird.
- [ ] Die Studierenden nutzen VR auf privaten VR-Brillen.
- [ ] Sonstiges:
- [ ] Das steht noch nicht fest.

Planen Sie, dass Studierende VR auch außerhalb der Lehrveranstaltung/en für das selbstbestimmte Lernen nutzen können?

?

- [ ] Ja, mit Leihgeräten
- [ ] Ja, in Räumlichkeiten unserer Einrichtung, in denen Hard- und Software bereitgestellt wird
- [ ] Ja, mit privaten VR-Brillen
- [ ] Nein
- [ ] Sonstiges:
- [ ] Das steht noch nicht fest.

4.3.2 Didaktische/technische Umsetzung (I)

Die folgenden Fragen beziehen sich auf Ihre **aktuelle Entwicklung von Lehrveranstaltungen** mit VR-Anteilen

Wie viele Studierende werden VR zukünftig pro Semester - mit Ihrer/n in Entwicklung befindlichen Lehrveranstaltung/en - an Ihrer Einrichtung nutzen? (circa)

Bitte geben Sie eine Zahl ein.

Werden Sie in Ihrer geplanten Lehrveranstaltung VR-Software verwenden, die nicht von oder mit Ihrer Einrichtung entwickelt wurde?

VR-kompatible Lernpakete, die mit 360°-Medien erstellt wurden, sind nicht gemeint.

- [ ] Ja, mit Nutzungsrechten einer kommerziellen Lizenz
- [ ] Ja, mit Nutzungsrechten einer CC-Lizenz
- [ ] Nein
- [ ] Sonstiges
- [ ] Das steht noch nicht fest.

Entwickelt Ihre Einrichtung die VR-Software für die geplante/n Lehrveranstaltung/en selbst oder ist sie an der Entwicklung beteiligt?

?

VR-kompatible Lernpakete, die mit 360°-Medien erstellt wurden, sind nicht gemeint.

- [ ] Ja, eigenständige VR-Software Entwicklung (inkl. 3D-Modellierung und Programmierung)
- [ ] Ja, Beteiligung mit medizinischem Fachwissen
- [ ] Ja, Beteiligung mit didaktischem Fachwissen
- [ ] Nein

4.3.2.1.1 Didaktische/technische Umsetzung (II)

Werden Sie Ihre VR-Software anderen Nutzer:innen zur Verfügung stellen?

?

- [ ] Ja, kommerziell
- [ ] Ja, kostenlos (z.B. unter einer CC-Lizenz)
- [ ] Nein
- [ ] Das steht noch nicht fest.

Mit welchen Einrichtungen und/oder Unternehmen arbeiten Sie bei der Entwicklung der VR-Software zusammen?

4.3.2.2 Kooperation (Ib)

Ist Ihr geplanter Einsatz von VR in der Lehre Teil oder Produkt eines geförderten Forschungs- und/ oder Entwicklungsprojekts?

- ◯ Ja
- ◯ Ja, zum Teil
- ◯ Nein

4.3.2.2.1.1 Kooperation (II)

Wie heißt oder hieß Ihr gefördertes VR-Projekt bzw. Ihre geförderten VR-Projekte?

?

Falls eine Projektwebsite vorhanden ist, bitte Link angeben.

Handelt/e es sich bei dem geförderten VR-Projekt bzw. den geförderten VR-Projekten um Kooperationsvorhaben?
Falls ja, wer ist/war an dem/den Vorhaben beteiligt?

- ◯ Ja, Beteiligte:
- ◯ Nein

5 Forschung (III)

Hat Ihre Einrichtung bereits wissenschaftliche Beiträge zu VR in der Lehre publiziert? Falls ja, welche?

Bitte geben Sie Links oder DOIs an.

Welche standortübergreifenden Austauschforen nutzen Sie im Bereich VR in der Lehre?

?

- ☐ Meetings im Drittmittelprojekt
- ☐ VR AG des GMA-Ausschusses für Digitalisierung
- ☐ Arbeitskreis VR/AR-Learning der Gesellschaft für Informatik
- ☐ UniVERSEty
- ☐ Sonstige:
- ☐ Keine

6 Abschluss

Nach dieser Seite gelangen Sie zum Ende des Fragebogens und können nicht mehr zurück.

Haben Sie noch weitere Anmerkungen zu Ihrem aktuellen oder geplanten Einsatz von VR in der Lehre?

z.B. zur technischen oder didaktischen Umsetzung

Wären Sie zu einem späteren Zeitpunkt bereit, in einem Online-Interview weitere Fragen zu Ihrem Einsatz von VR in der Lehre zu beantworten?

Falls ja, kommen wir gerne im Verlauf des Sommers 2023 auf Sie zu.

- ○ Ja
- ○ Nein

7 Endseite

Vielen Dank für Ihre Teilnahme an der Befragung!

Haben Sie noch Fragen oder Feedback? Dann wenden Sie sich bitte an:

Robert Speidel
Medizinische Fakultät Ulm
Kompetenzzentrum eEducation in der Medizin BW
Robert.Speidel@uni-ulm.de
+49 (0)731 50 33685
